# RNA-Seq quantification of the human small airway epithelium transcriptome

**DOI:** 10.1186/1471-2164-13-82

**Published:** 2012-02-29

**Authors:** Neil R Hackett, Marcus W Butler, Renat Shaykhiev, Jacqueline Salit, Larsson Omberg, Juan L Rodriguez-Flores, Jason G Mezey, Yael Strulovici-Barel, Guoqing Wang, Lukas Didon, Ronald G Crystal

**Affiliations:** 1 Department of Genetic Medicine, Weill Cornell Medical College, New York, New York, USA; 2 Department of Biological Statistics and Computational Biology, Cornell University, Ithaca, New York, USA

## Abstract

**Background:**

The small airway epithelium (SAE), the cell population that covers the human airway surface from the 6^th ^generation of airway branching to the alveoli, is the major site of lung disease caused by smoking. The focus of this study is to provide quantitative assessment of the SAE transcriptome in the resting state and in response to chronic cigarette smoking using massive parallel mRNA sequencing (RNA-Seq).

**Results:**

The data demonstrate that 48% of SAE expressed genes are ubiquitous, shared with many tissues, with 52% enriched in this cell population. The most highly expressed gene, SCGB1A1, is characteristic of Clara cells, the cell type unique to the human SAE. Among other genes expressed by the SAE are those related to Clara cell differentiation, secretory mucosal defense, and mucociliary differentiation. The high sensitivity of RNA-Seq permitted quantification of gene expression related to infrequent cell populations such as neuroendocrine cells and epithelial stem/progenitor cells. Quantification of the absolute smoking-induced changes in SAE gene expression revealed that, compared to ubiquitous genes, more SAE-enriched genes responded to smoking with up-regulation, and those with the highest basal expression levels showed most dramatic changes. Smoking had no effect on SAE gene splicing, but was associated with a shift in molecular pattern from Clara cell-associated towards the mucus-secreting cell differentiation pathway with multiple features of cancer-associated molecular phenotype.

**Conclusions:**

These observations provide insights into the unique biology of human SAE by providing quantit-ative assessment of the global transcriptome under physiological conditions and in response to the stress of chronic cigarette smoking.

## Background

The tracheobronchial tree, a dichotomous branching structure that begins at the larynx and ends after 23 branches at the alveoli, is lined by an epithelium comprised of 4 major cell types, including ciliated, secretory, undifferentiated columnar and basal cells [1,2]. The airway epithelium is exposed directly to environmental xenobiotics, particulates, pathogens and other toxic substances suspended in inhaled air [2-4]. Of these, chronic cigarette smoking, with its 4000 xenobiotics and > 10^14 ^oxidants per puff, is a major cause of airway disease, including chronic obstructive pulmonary disease (COPD) and bronchogenic carcinoma [4-6]. It is the airway epithelium that exhibits the first abnormalities relevant to COPD and lung cancer, and it is the small airway epithelium (SAE; ≥ 6^th ^generation) that is the primary site of the early manifestations of the majority of smoking-induced lung disease [7]. As compared to proximal airways, the small airway epithelium has unique morphologic features with a decrease in the frequency of basal cells and mucus-secreting cells accompanied by increased numbers of Clara cells, a secretory cell subtype critical for the maintenance of the structural and functional integrity at the airway-alveoli interface [1,8-10].

Our group [11-13] and others [14-18] have carried out several studies using gene expression microarrays to assess the transcriptome of the human airway epithelium, demonstrating that smoking modulates the expression of hundreds of genes. The advent of RNA-Seq technology, in which the entire polyadenylated transcriptome is sequenced [19-24], is capable of building on this microarray data to provide additional insights into the transcriptome of the airway epithelium and its response to cigarette smoke. Because RNA-Seq provides direct sequencing information of all polyadenylated mRNAs and is not limited by probe design, RNA-Seq data has inherently less noise and higher specificity, and, importantly, provides quantitative information on mRNA transcript number [19]. With high sensitivity and low background, RNA-Seq has a dynamic range of > 8,000-fold, is highly reproducible, yields digital information not requiring normalization, and can distinguish individual members of highly homologous gene families [25]. In the context of this background, the focus of this study is to utilize massive parallel sequencing to quantify the complete transcriptome of the human SAE in healthy nonsmokers and healthy smokers.

## Results

### Study Population and SAE sampling

SAE samples from 5 healthy nonsmokers and 6 healthy smokers were analyzed using mRNA-Seq (Additional file 1, Table S1). All individuals had no significant prior medical history and a normal physical examination. To minimize the influence of potential confounding variables, only males of African-American ancestry were assessed. The nonsmokers were younger (p < 0.02). There were no differences be-tween the two groups with respect to pulmonary function criteria (p > 0.1, all variables). The smoking status was confirmed by urinary tobacco metabolites (see Additional Data Methods and Additional file 1, Table S1). The number of cells recovered ranged from 5.0 to 9.7 × 10^6^, with > 97% epithelial cells in all cases (Additional file 1, Table S1). There was no difference between the two groups with respect to the proportions of each of the four major cell types, with the exception of ciliated cells, which were significantly lower in the healthy smokers compared to the healthy nonsmokers (p < 0.04).

### Data Processing and Quality Control

**Figure 1:**
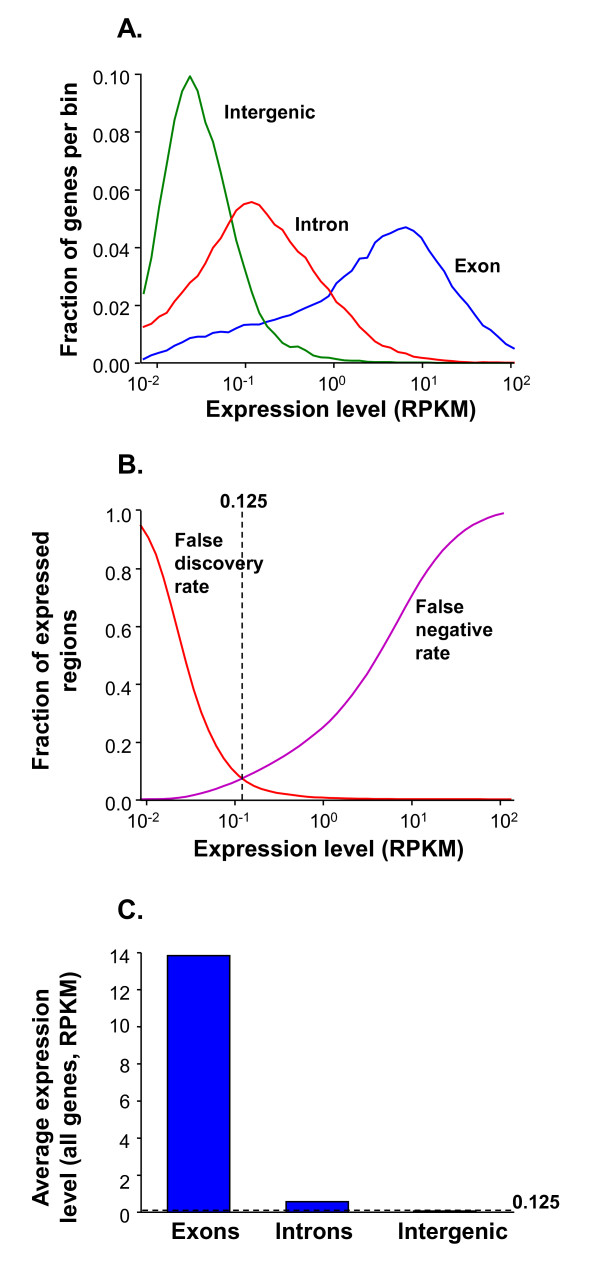
**Establishment of detection limit for gene expression for RNA-Seq assessment of gene expression of the small airway epithelium of healthy nonsmokers**. **A**. Distribution of RPKM for exons (blue), introns (red), and intergenic regions (green). RPKM depends on the size and read numbers mapped in the region considered. The RPKM for introns and intergenic regions was calculated by selecting intronic and intergenic regions throughout the genome that match the size of the exons analyzed, i.e., the size is comparable for the introns, intergenic regions, and exons. **B**. Estimate of minimum detectable level of expression (RPKM = 0.125) determined from an estimate of false discovery rate (red) and false negative rate (purple) [24]. **C**. Average RPKM expression levels (log_10_). Dashed line represents the 0.125 threshold.

The cDNA generated from SAE samples was run in a single lane per subject on Illumina flow cells. A total of 182 million, 43 base pair single end reads were generated, yielding 7.8 gigabases of sequence. These sequences were aligned using Bowtie version 0.12 (see Additional file 1, Table S2 for a summary of mapping details). To correct for transcript length and depth of coverage, raw reads were converted into reads per kilobase of exon per million mapped reads (RPKM) [23]. RPKM was then assessed across the entirety of reads with reference to exons, introns and intergenic regions. A comparison was made between the expression levels of exons and intergenic regions to define a threshold value above which there was the highest confidence in the validity of the expression value (Figure 1A, B). This was performed by generating a false discovery rate for a range of expression values across all subjects, resulting in the adoption of a cut-off value of 0.125 RPKM, representing an optimal compromise between false positive and false negative values (see Methods). All subsequent analyses were based on the application of this expression threshold.

Of the 21,475 annotated genes in the Human Genome version 19 reference [26], 15,877 (73.9%) were expressed in SAE at greater than the RPKM cut-off value of 0.125. The average expression level was 13.8 RPKM (Figure 1C) and the average among subject coefficient of variation in RKPM was 0.25. This cut off may be conservative due to overestimation of the number of intronic and intergenic reads. Based on a survey of a random intergenic domain on the genome, we estimate that ~50% of the reads mapped to intergenic and intronic regions correspond to repetitive elements. These probably represent mismapping of reads that properly belong polyadenylated mRNAs that contain the same repetitive elements. If the impact was to drop the threshold from 0.125, as used here, to 0.05, the number of expressed genes would increase from 15,877 to 16,844 (a 6.1% increase). It is estimated that 1 RPKM corresponds to one mRNA per cell. As a result, we believe there is little significant biology lost by using a conservative cut off (consistent with literature precedent) of 0.125 (i.e., one mRNA per 8 cells) *vs *a more stringent cut off of 0.05 (one mRNA per 20 cells).

A subset of 12 genes with RPKM differing by > 4 logs was confirmed by TaqMan realtime quantitative PCR with relative quantitation, using rRNA for normalization. The overall correlation between expression levels determined by these 2 methods was very strong (r^2 ^= 0.81) (Additional file 1, Figure S1).

### Comparison of SAE Gene Expression to Other Tissue Transcriptomes

**Figure 2:**
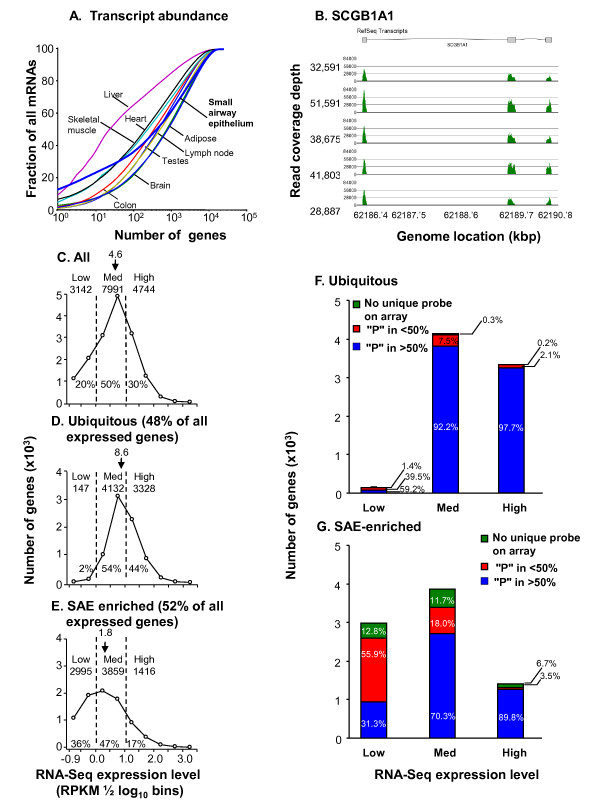
**Composition of the healthy nonsmoker small airway epithelium (SAE) transcriptome**. **A**. Comparison of the SAE transcriptome to that of other tissues. Abscissa - the number of genes, with the genes in descending order of mRNA level. Ordinate - fraction of all mRNAs derived from these genes. The genes expressed by the small airway epithelium (blue) are compared to genes expressed by other organs as indicated [24]. Note that the SAE is similar to liver in that a few genes are expressed at very high levels. **B**. RNA-Seq sequence alignments for SCGB1A1 (uteroglobin; CC10), the most highly expressed gene in the SAE. The region of the genome corresponding to SCGB1A1 is shown with the read coverage depth for 5 healthy nonsmokers plotted using Partek Genomics Suite version 6.5. RPKM for whole mRNA for each subject is shown on the left. **C-E**. Frequency distribution of expression level for ubiquitous *vs *SAE-enriched genes in the small airway epithelium of healthy nonsmokers. "Ubiquitous" genes are those expressed by most tissues; "SAE-enriched" genes are those more abundant in SAE compared to other tissues (see text). For all panels, the number of genes in 1/2 log_10 _bins was determined starting at the detection limit (RPKM = 0.125). For each panel, the expressed genes are grouped (in 1/2 log_10 _bins); low (-0.9 to 1), median (> 1 to 10) and high (> 10), with the number of genes and % in each category listed and median RPKM for n = 5 healthy nonsmokers. **C**. All genes. **D**. Ubiquitous genes, representing 48% of all expressed genes. **E**. SAE-enriched genes representing 52% of all expressed genes. Note that the SAE-enriched genes have a much larger proportion of low level expressed genes compared to the ubiquitous genes. **F, G**. Comparison of coverage of RNA-Seq and microarray assessment of SAE gene expression of healthy nonsmokers. Genes assessed by RNA-Seq were divided into low (0.125-1), median (> 1-10) and high (> 10) RPKM on the basis of median expression level in n = 5 nonsmokers. Affymetrix U133 data for small airway epithelium for n = 27 African-American healthy nonsmokers [[129]; Additional file 1, Table S1] were assessed based on the Affymetrix P calls in low expression (Affymetrix "present (P)" in < 50%; red) or high expression in microarray ("P" in > 50%; blue). Genes with no unique probe on the microarray are identified in green. **F**. Ubiquitous genes. **G**. SAE- enriched genes. For medium and high expressing genes the microarray and RNA-Seq are very similar in detecting expressed genes, but for the SAE-enriched, low level expressed genes detected by RNA-Seq, the microarrays miss a large proportion of the genes.

RNA-Seq allows absolute quantitation of mRNA levels and for the fractional contribution of individual transcripts to the total mRNA population to be assessed [24]. In some tissues, transcripts from a relatively small number of genes account for much of the total cellular poly(A)^+ ^RNA pool (Figure 2A). In the case of liver, the single most highly expressed gene contributes 10% of the total mRNA molecules and the top ten collectively contribute 37% [24]. In colon, by contrast, the single top mRNA contributes only 2% of the total mRNA and the top ten contribute 9%[24]. Of the total SAE transcripts identified in healthy nonsmokers, 13% mapped to the SCGB1A1 gene (secretoglobin, family 1A, member 1 protein also known as ute-roglobin or Clara cell-specific 10 kD protein [CC10], Table 1). The top 10 genes contributed 24% of the total mRNA (p < 0.05 comparing distribution to both liver, and colon).

**Table 1:** Overall Most Highly Expressed Genes in the SAE of Healthy Nonsmokers^1^

Gene symbol	Gene title	**Expression level in SAE (RPKM)**^**2**^
SCGB1A1	secretoglobin, family 1A, member 1 (uteroglobin)	38675
SCGB3A1	secretoglobin, family 3A, member 1	7838
SLPI	secretory leukocyte peptidase inhibitor	1602
C20orf114	chromosome 20 open reading frame 114	1484
TPPP3	tubulin polymerization-promoting protein family member 3	1302
CD74	CD74 molecule, major histocompatibility complex, class II invariant chain	947
TMEM190	transmembrane protein 190	945
GSTP1	glutathione S-transferase pi 1	859
WFDC2	WAP four-disulfide core domain 2	840
C20orf85	chromosome 20 open reading frame 85	738
TSPAN1	tetraspanin 1	664
C9orf24	chromosome 9 open reading frame 24	629
NEAT1	nuclear paraspeckle assembly transcript 1 (non-protein coding)	565
S100A11	S100 calcium binding protein A11	540
KRT19	keratin 19	493
MALAT1	metastasis associated lung adenocarcinoma transcript 1 (non-protein coding)	461
ODF3B	outer dense fiber of sperm tails 3B	392
CYP4B1	cytochrome P450, family 4, subfamily B, polypeptide 1	374
FOXJ1	forkhead box J1	363
LCN2	lipocalin 2	359
PIGR	polymeric immunoglobulin receptor	351
MS4A8B	membrane-spanning 4-domains, subfamily A, member 8B	348
ALDH3B1	aldehyde dehydrogenase 3 family, member B1	342
MSMB	microseminoprotein, beta-	333
RSPH1	radial spoke head 1 homolog (Chlamydomonas)	318
CLDN4	claudin 4	308
AQP3	aquaporin 3 (Gill blood group)	308
C9orf117	chromosome 9 open reading frame 117	302
IGFBP2	insulin-like growth factor binding protein 2, 36 kDa	297
ANXA2P2	annexin A2 pseudogene 2	292

^1 ^Listed are the top 30 most highly expressed SAE enriched genes.

^2 ^Median for n = 5 healthy nonsmokers. PARTEK implementation of Bowtie algorithm with parameters as described in Material and Methods.

### Ubiquitous and SAE-enriched Genes

To categorize the SAE-expressed genes in healthy nonsmokers as ubiquitous (i.e., expressed by most other tissues) or genes expressed in an SAE-enriched fashion, a comparison was made between the 7,897 genes identified by Ramsköld et al [24] to be ubiquitously expressed in various human tissues and the 15,877 genes expressed in human SAE. The data showed that 7,607 (96.5%) of the genes identified by Ramsköld et al [24] as ubiquitously expressed genes were also expressed by human SAE, indicating that 48% of the SAE transcriptome is comprised of ubiquitously expressed genes. The remaining 52% were designated as "SAE-enriched" genes.

The most highly expressed SAE-enriched gene (Table 1) was SCGB1A1, which is expressed primarily by Clara cells located in small airways [27-29]. RNA-Seq fragments mapped to all three exons of the SCGB1A1 gene at very high density (Figure 2B). Other highly expressed SAE-enriched genes included secretoglobin, family 3A, member 1(SCGB3A1), secretory leukocyte peptidase inhibitor (SLPI), chromosome 20 open reading frame 114 (C20orf114; also known as a long variant of the palate, lung, and nasal epithelium carcinoma associated protein PLUNC), tubulin polymerization-promoting protein family member 3 (TPPP3) and CD74.

To further characterize the SAE transcriptome, gene expression levels derived from RNA-Seq data were divided into three groups. "Low" expression was assigned as significantly expressed (i.e., > 0.125 RPKM cut off, but less than 1 RPKM, corresponding to < 1 mRNA/cell [23]). "Medium" expression was defined at between 1 and 10 RPKM and "high" expression was defined as > 10 RPKM (Figure 2C-E). Analyses of the frequency distribution of ubiquitous and SAE-enriched RefSeq-annotated genes revealed that considerably more of the SAE-enriched gene set were expressed at lower levels (median expression level 1.8 RPKM, Figure 2E) compared to the ubiquitous genes (median expression level 8.6 RPKM, Figure 2D).

Prior to the advent of RNA-Seq method, information about the transcriptome of airway epithelium was derived from gene expression microarrays [11-18]. To assess the concordance of expression pattern by RNA-Seq and microarrays, all genes expressed by RNA-Seq were evaluated as to whether they were identified as expressed in all microarrays, a subset of microarrays or not represented on the microarray (Figures 2F, G). For ubiquitous genes, the percentage of genes identified by RNA-Seq also identified as expressed in > 50% of subjects by microarray was greater for highly expressed genes (97.7%) than medium expressed genes (92.1%). Only 59.2% of ubiquitous genes with low expression identified by RNA-Seq were scored as expressed by microarray (Figure 2F). Similarly, for the SAE-enriched genes, the percent of genes identified by RNA-Seq and in > 50% of subjects by microarray was greater for highly expressed genes (89.8%), compared to medium expressed genes (70.3%), and even more so for genes with low expression (31.3%; Figure 2G). Thus, overall, the two methods are broadly in agreement, but RNA-Seq provides more information, not only quantitative data, but also identification of expression of genes with low expression levels.

### Functional Assessment of Ubiquitous and SAE-enriched Genes

**Figure 3:**
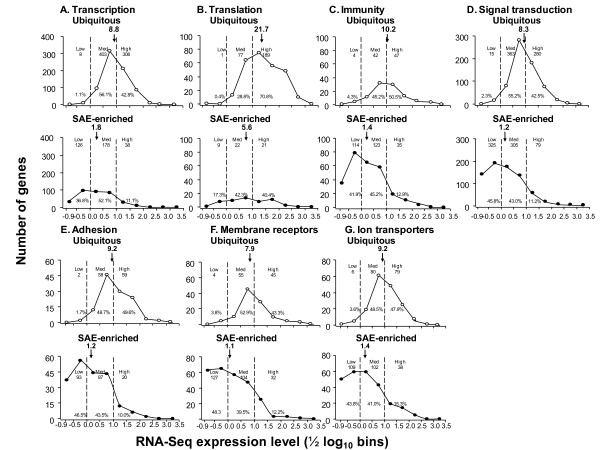
**Relative distribution of the expression of ubiquitous and SAE-enriched genes of healthy non-smokers in different functional categories**. Gene ontology assignments were used to identify genes of 7 functional categories and the frequency distribution of expression level was determined in 1/2 log_10 _bins, starting with the threshold (RPKM 0.125 = -0.9 log_10_). The data is plotted separately for the ubiquitous genes (open symbols) and SAE-enriched genes (closed symbols) with the number and percentage of genes in each low, medium and high group. For each panel, for each group, listed is the number of genes, % of the total in that category, and median RPKM for n = 5 healthy nonsmokers (number with downward arrow). **A**. Transcription; **B**. Translation; **C**. Immunity; **D**. Signal transduction; **E**. Adhesion; **F**. Membrane receptors; and **G**. Ion transporters.

To better understand biologic functions enriched in the SAE transcriptome, the gene lists were assigned to functional categories using Gene Ontology molecular functions and the expression levels for ubiquitous and SAE-enriched genes were compared (Figure 3, Additional file 1, Table S3). In some functional categories such as signal transduction, the allotment of genes to the SAE-enriched and ubiquitous categories, as well as the distribution of expression levels within those categories, was similar to that for all genes (compare Figure 3D to Figures 2D, E). But several deviations from the expected distribution were observed. For example, for the functional category "translation", many more genes were classified as ubiquitous compared to SAE-enriched and those that were SAE-enriched were expressed at higher levels than expected based on all genes. By contrast, in the category "immunity", the number of genes in the SAE-enriched category was higher than expected but distribution of expression level was in line with the average expression of all genes. The median expression levels among the various categories allowed quantification of these differences. The median expression levels for the SAE-enriched genes were lower than that for the ubiquitous genes in all categories. For example, for the ubiquitous genes, the median levels ranged from 7.9 RPKM for membrane receptors to 21.7 RPKM for translation, whereas, for the SAE-enriched genes, the median levels ranged from 1.1 for membrane receptors to 5.6 RPKM for translation. On the average, the most highly expressed category was "translation, ubiquitous" genes (median 21.7 RPKM), whereas the lowest was "membrane receptors, SAE-enriched" (median 1.1 RPKM).

**Figure 4:**
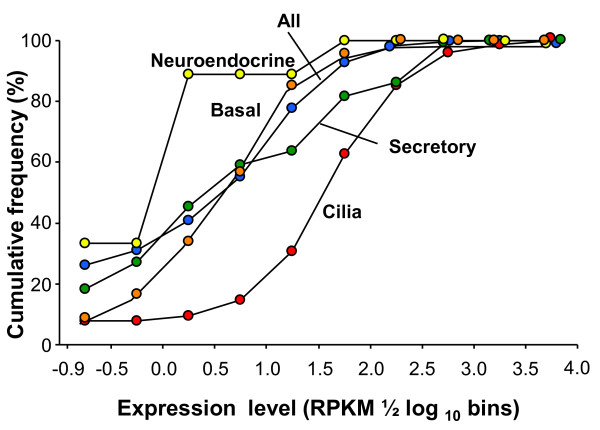
**Distribution of expression level of cell type-specific genes in the small airway epithelium of healthy nonsmokers**. Lists of genes specific to neuroendocrine cells, basal cells, secretory cells (including all mucins) and ciliated cells were used to assess the cumulative frequency distribution of expression levels for each category. The lists of cell type-specific genes are from the literature, including neuroendocrine genes [31], basal cells [30], secretory cells and ciliated cells [52]. Ordinate - cumulative frequency; abscissa - expression level (RPKM in 1/2 log_10 _bins).

The human SAE is made up of 4 major cell types including ciliated cells (73% abundance in this study), undifferentiated columnar cells (9%), basal cells (10%) and secretory cells (7%, Additional file 2, Table S1). The SAE also has rare neuroendocrine cells (< 0.01%) [13,30,31]. The availability of cell type-specific gene lists, together with the ability of RNA-Seq to quantify mRNA abundance, allows the contributions of these cell types to the SAE transcriptome to be assessed quantitatively. When compared to all genes of the SAE-enriched transcriptome, genes encoding cilia components were expressed at much higher levels and genes encoding neuroendocrine cell genes were expressed at much lower levels (Figure 4). On the other hand, genes identified as representative of the basal cells and secretory cells were expressed at levels comparable with the average level for SAE-enriched genes. The highest expressed cilia-related genes included tubulin β2C and α1A subunits, tektin and a number of dynein subunits with RPKM of > 100 (Table 2). By contrast, neuroendocrine genes such as secreogranin II (SCG2) and chromogranin A (CHGA) were expressed at < 1 RPKM with the exception of enolase 2, which may not be neuroendocrine cell-specific [32]. Among the mucus-secreting cell genes, trefoil factor 3 and two mucin genes, MUC1 and MUC5B, were the most highly expressed (RPKM > 100). For the basal cell genes, when assessed in the context of all SAE genes, MALAT1 (a noncoding transcript), CST3 cystatin C (a protease inhibitor) and PFN1 profilin 1 (a ubiquitous actin monomer-binding protein) were the most highly expressed.

**Table 2:** Most Highly Expressed Genes Enriched in Differentiated Cell Types of the SAE of Healthy Nonsmokers^1^

Differentiated cell type	Gene symbol	Gene title	**Expression level in SAE (RPKM)**^**2**^
**Ciliated**	TUBB2C	tubulin, beta 2C	1161
	ACTG1	actin, gamma 1	513
	TUBA1A	tubulin, alpha 1a	342
	HSPA1A	heat shock 70 kDa protein 1A	266
	HSPA1B	heat shock 70 kDa protein 1A///heat shock 70 kDa protein 1B	260
	TEKT2	tektin 2 (testicular)	146
	DYNLT1	dynein, light chain, Tctex-type 1	131
	DNAI1	dynein, axonemal, intermediate chain 1	108
	DNALI1	dynein, axonemal, light intermediate chain 1	108
	DNAI2	dynein, axonemal, intermediate chain 2	105
	SPAG6	sperm associated antigen 6	101
	DYNLRB2	dynein, light chain, roadblock-type 2	100
	CROCC	ciliary rootlet coiled-coil, rootletin	84
	PPP2R1A	protein phosphatase 2, regulatory subunit A, alpha	73
	DNAH9	dynein, axonemal, heavy chain 9	63
	CCDC146	coiled-coil domain containing 146	60
	RSPH4A	radial spoke head 4 homolog A (Chlamydomonas)	59
	CALM3	calmodulin 3 (phosphorylase kinase, delta)	56
	TCTEX1D2	Tctex1 domain containing 2	50
	IFT140	intraflagellar transport 140 homolog (Chlamydomonas)	49
**Mucins and mucus components**	AGR2	anterior gradient homolog 2	166
	TFF3	trefoil factor 3 (intestinal)	149
	MUC1	mucin 1, cell surface associated	123
	MUC5B	mucin 5B, oligomeric mucus/gel-forming	118
	MUC4	mucin 4, cell surface associated	93
	MUC15	mucin 15, cell surface associated	28
	MUC20	mucin 20, cell surface associated	27
	MUC16	mucin 16, cell surface associated	20
	MUC13	mucin 13, cell surface associated	15
	MUCL1	mucin-like 1	3.90
	TFF1	trefoil factor 1	2.09
	PARM1	prostate androgen-regulated mucin-like protein 1	1.85
	EMR2	egf-like module containing, mucin-like, hormone receptor-like 2	1.38
	GCNT3	glucosaminyl (N-acetyl) transferase 3, mucin type	0.81
	MUC2	mucin 2, oligomeric mucus/gel-forming	0.60
	MUC6	mucin 6, oligomeric mucus/gel-forming	0.58
	MUC12	mucin 12, cell surface associated	0.46
**Basal**	MALAT1	metastasis associated lung adenocarcinoma transcript 1 (non-protein coding)	461
	CST3	cystatin C	295
	PFN1	profilin 1	224
	ALDOA	aldolase A, fructose-bisphosphate	183
	SQSTM1	sequestosome 1	106
	MT2A	metallothionein 2A	91
	ENO1	enolase 1, (alpha)	89
	KRT7	keratin 7	83
	MYL12A	myosin, light chain 12A, regulatory, non-sarcomeric///myosin, light chain 12B, regulatory	70
	FLNB	filamin B, beta	69
	BRI3	brain protein I3///hypothetical protein LOC644975	62
	PLEC1	plectin	60
	EIF5A	eukaryotic translation initiation factor 5A	60
	GNB1	guanine nucleotide binding protein (G protein), beta polypeptide 1	57
	KRT5	keratin 5	54
	PSMA7	proteosome subunit alpha type 7	53
	CTTN	cortactin	52
	JUP	junction plakoglobin	51
	MGST1	microsomal glutathione S-trasnferase	51
	LMNA	laminin A	49
**Neuro- endocrine**	ENO2	enolase 2 (gamma, neuronal)	23
	GRP	gastrin-releasing peptide	0.82
	UCHL1	ubiquitin carboxyl-terminal esterase L1 (ubiquitin thiolesterase)	0.65
	SCG2	secretogranin II	0.48
	ASCL1	achaete-scute complex homolog 1 (Drosophila)	0.34
	CHGA	chromogranin A (parathyroid secretory protein 1)	0.32

^1 ^List includes genes known to be enriched in expression in ciliated cells (Dvorak et al. 2010) [52], secretory cells (consisting of all mucins and mucin components), basal cells (Hackett et al. 2011) [30], and neuroendocrine cells (Carolan et al. 2008) [31]. The small airway epithelium expression level was determined and the top 20 highly expressed (or all genes expressed from mucin list and neuroendocrine list, i.e. above threshold of 0.125) were tabulated in descending order of expression level.

^2 ^Median for n = 5 nonsmokers.

To obtain insight into transcriptional regulation of the SAE of nonsmokers, the SAE-enriched transcriptome was surveyed for the most highly expressed transcription factors in various structural categories (Table 3). Among the top 30 most highly expressed, the helix-turn-helix dominated, with the basic helix-loop-helix and β-scaffold categories next. Interestingly, the top 5 most highly expressed SAE-enriched transcription factors all have previously been established as having a role in airway biology and/or lung cancer, including FOXJ1, ELF3, TRIM29, SOX2, and FOXA1 [33-43]. RNA-Seq analysis also revealed high expression levels for a variety of pathway-specific transcription factors, including two (HES6, HEY1) related to notch signaling.

**Table 3:** Highly Expressed SAE-enriched Transcription Factors^1^

Category	Gene symbol	Gene title	Median expression level (RPKM)
Basic helix-loop -helix	ATF6B	activating transcription factor 6 beta	26.4
	BHLHE40	basic helix-loop-helix family, member e40	16.2
	RFX3	regulatory factor X, 3 (influences HLA class II expression)	15.6
	HES6	hairy and enhancer of split 6 (Drosophila)	13.6
	FOXC1	forkhead box C1	9.3
	CEBPA	CCAAT/enhancer binding protein (C/EBP), alpha	9.1
	HEY1	hairy/enhancer-of-split related with YRPW motif 1	9.1
Zinc finger	TRIM29	tripartite motif-containing 29	77.3
	KLF5	kruppel-like factor 5 (intestinal)	42.0
	RREB1	ras responsive element binding protein 1	7.2
	KLF4	kruppel-like factor 4 (gut)	7.1
Helix-turn-helix	FOXJ1	forkhead box J1	363.1
	ELF3	E74-like factor 3 (ets domain transcription factor, epithelial-specific)	170.1
	FOXA1	forkhead box A1	62.6
	EHF	ets homologous factor	39.1
	TBX1	T-box 1	19.7
	SATB1	SATB homeobox 1	19.3
	MYB	v-myb myeloblastosis viral oncogene homolog (avian)	15.9
	SIX2	SIX homeobox 2	13.9
	NKX2-1	NK2 homeobox 1	11.6
	PHTF1	putative homeodomain transcription factor 1	10.9
	TEAD3	TEA domain family member 3	10.9
	ETV6	ets variant 6	10.6
	FOXA2	forkhead box A2	6.9
β-scaffold	SOX2	SRY (sex determining region Y)-box 2	71.3
	RUNX1	runt-related transcription factor 1	16.7
	SOX4	SRY (sex determining region Y)-box 4	11.4
	TFCP2	transcription factor CP2	10.7
	SOX21	SRY (sex determining region Y)-box 21	9.0
	NFATC1	nuclear factor of activated T-cells, cytoplasmic, calcineurin-dependent 1	7.8
	SOX9	SRY (sex determining region Y)-box 9	7.7

^1 ^The top 30 DNA-binding transcription factors were identified in the highly expressed SAE-enriched list for n = 5 healthy nonsmokers. They were categorized by transcription factor family and sorted in descending order of expression within each category.

To quantify the expression of the receptors and ligands that may be involved in epithelial maintenance, the most highly expressed transmembrane receptors in different structural categories were identified (Table 4). Discoidin domain receptor tyrosine kinase 1 (DDR1), a collagen receptor associated with poor prognosis in non-small cell lung cancer, was the most highly expressed transmembrane receptor [44]. There were a significant number of G protein coupled, 7 transmembrane receptors in the highly expressed category, including a homophilic cadherin-coupled receptor (CELSR1) and the complement 5a receptors, as well as 2 orphan receptors (GPR110, GPRC5C). In addition to DDR1, there were also a number of highly expressed tyrosine kinase receptors among the top 30, including fibroblast growth factor receptors (FGFR3, FGFR2) and the insulin like growth factor 1 receptor (IGF1R). Interestingly, the receptors for oxytocin and natriuretic peptide were also expressed at a high level.

**Table 4:** Highly Expressed SAE-enriched Transmembrane Receptors^1^

Category	Gene symbol	Gene title	Median expression level (RPKM)
G protein coupled/7 transmembrane	CELSR1	cadherin, EGF LAG seven-pass G-type receptor 1 (flamingo homolog, Drosophila)	63.0
	C5AR1	complement component 5a receptor 1	27.6
	GPR110	G protein-coupled receptor 110	21.7
	GPRC5C	G protein-coupled receptor, family C, group 5, member C	20.3
	OXTR	oxytocin receptor	19.3
	LPAR3	lysophosphatidic acid receptor 3	17.6
	FZD6	frizzled homolog 6 (Drosophila)	12.5
	PTGER4	prostaglandin E receptor 4 (subtype EP4)	9.2
	VIPR1	vasoactive intestinal peptide receptor 1	8.8
	ADRB1	adrenergic, beta-1-, receptor	7.8
	GPR116	G protein-coupled receptor 116	7.5
	FZD8	frizzled homolog 8 (Drosophila)	7.1
	ADRA2A	adrenergic, alpha-2A-, receptor	6.8
	PTGFR	prostaglandin F receptor (FP)	6.6
Cyclase related	NRP2	neuropilin 2	12.0
	NPR2	natriuretic peptide receptor B/guanylate cyclase B (atrionatriuretic peptide receptor B)	10.7
	CRCP	CGRP receptor component	10.4
IgG like	SCARA3	scavenger receptor class A, member 3	21.8
	PTPRT	protein tyrosine phosphatase, receptor type, T	13.0
	IL1R1	interleukin 1 receptor, type I	6.5
Ion channel	GABRP	gamma-aminobutyric acid (GABA) A receptor, pi	15.7
Serine kinase	TGFBR2	transforming growth factor, beta receptor II (70/80 kDa)	8.8
Tyrosine kinase	DDR1	discoidin domain receptor tyrosine kinase 1	140.3
	FGFR3	fibroblast growth factor receptor 3	19.9
	IGF1R	insulin-like growth factor 1 receptor	15.2
	PTK7	PTK7 protein tyrosine kinase 7	14.5
	FGFR2	fibroblast growth factor receptor 2	11.6
	MET	met proto-oncogene (hepatocyte growth factor receptor)	8.5
	EGFR	epidermal growth factor receptor (erythroblastic leukemia viral (v-erb-b) oncogene homolog, avian)	7.2
Other	SORL1	sortilin-related receptor, L(DLR class) A repeats-containing	7.7

^1 ^The top 30 transmembrane receptors were identified in the highly expressed SAE-enriched list for n = 5 healthy nonsmokers. They were categorized by structural family and sorted in descending order of expres-sion within each category.

With respect to ligands and growth factors, the most highly expressed included multiple chemokines MDK, CXCL1, CX3CL1 and CXCL6 (Table 5). No known ligand of the top 10 most highly expressed receptors was expressed at RPKM > 5 in the SAE-enriched transcriptome, nor was any known receptor for the top 10 highly expressed ligands expressed in the SAE-enriched transcriptome at RPKM > 5. This observation is of interest, as it suggests that much of the biology of the SAE relates to interactions of the SAE as target (receptors) or source (ligands) of external stimuli modulating SAE function or *vice versa*.

**Table 5:** Highly Expressed SAE-enriched Signaling Ligands and Growth Factors^1^

Gene symbol	Gene title	Median expression level (RPKM)
MDK	midkine (neurite growth-promoting factor 2)	59.8
CXCL1	chemokine (C-X-C motif) ligand 1 (melanoma growth stimulating activity, alpha)	49.3
CX3CL1	chemokine (C-X3-C motif) ligand 1	32.9
TNFSF10	tumor necrosis factor (ligand) superfamily, member 10	31.8
FSTL1	follistatin-like 1	27.9
CXCL6	chemokine (C-X-C motif) ligand 6 (granulocyte chemotactic protein 2)	18.6
FGF14	fibroblast growth factor 14	16.5
DLL1	delta-like 1 (Drosophila)	13.6
JAG2	jagged 2	11.9
IL8	interleukin 8	8.6
CCL15	C-C motif chemokine 15	7.8
PDGFA	platelet-derived growth factor alpha polypeptide	6.1
TNFSF12	tumor necrosis factor (ligand) superfamily, member 12	5.9
CCL23	chemokine (C-C motif) ligand 23	5.3
NMB	neuromedin B	4.9
CCL5	chemokine (C-C motif) ligand 5	4.9
NPFF	neuropeptide FF-amide peptide precursor	4.6
FAS	fas (TNF receptor superfamily, member 6)	3.6
WIF1	WNT inhibitory factor 1	3.6
CCL18	chemokine (C-C motif) ligand 18 (pulmonary and activation-regulated)	3.5
LTB	lymphotoxin beta (TNF superfamily, member 3)	3.0
LIF	leukemia inhibitory factor (cholinergic differentiation factor)	2.9
ERBB4	v-erb-a erythroblastic leukemia viral oncogene homolog 4 (avian)	2.7
PTCH1	patched homolog 1 (Drosophila)	2.6
WNT5A	wingless-type MMTV integration site family, member 5A	2.1
CCL17	chemokine (C-C motif) ligand 17	1.7
NRTN	neurturin	1.7
AREG	amphiregulin	1.4
CCL4	chemokine (C-C motif) ligand 4	1.3
NTS	neurotensin	1.2

^1 ^The top 30 signaling ligands and growth factors were identified in the highly expressed SAE-enriched list for n = 5 healthy nonsmokers.

### Gene Families

**Figure 5:**
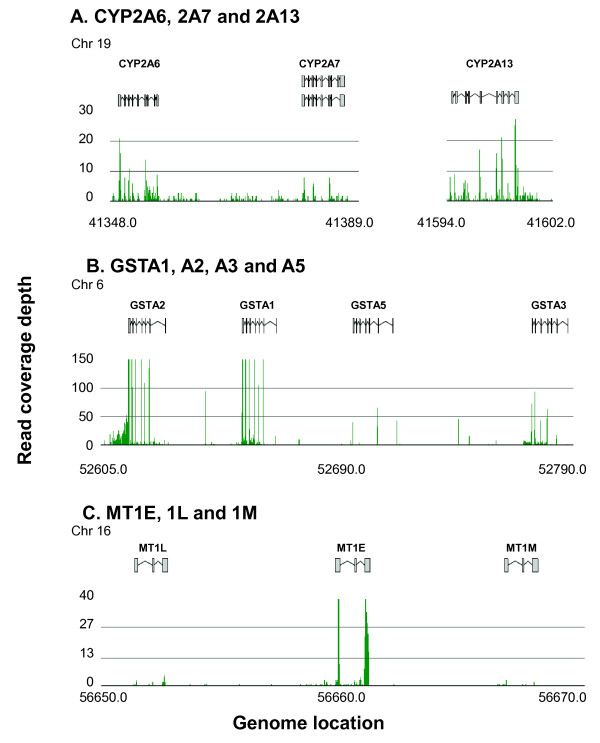
**Examples of RNA-Seq quantification of small airway epithelium (SAE) expression levels of genes within gene families of ≥ 90% homology**. To identify gene families expressed by the SAE, the % identity between gene pairs expressed by the healthy nonsmoker SAE was determined using BLAST, where each gene was blasted against a database of all human RefSeq mRNA [26]. Gene families were defined as genes for which the alignments yielded ≥ 90% identity and the alignment length was at least 50% of both sequences. **A**. CYP2A6, CYP2A7 and CYP2A13; **B**. GSTA1, GSTA2, GSTA3 and GSTA5; and **C**. MT1E, MT1L and MT1M.

One advantage of RNA-Seq compared to microarray is that transcripts can be unequivocally mapped to a single member of a gene family when sequence is similar but not identical. Thus, RNA-Seq can be used to identify and quantify highly homologous genes, something not possible with hybridization-based microarrays [25]. To quantify SAE expression of homologous gene families in healthy nonsmokers, we identified all gene pairs with ≥ 90% sequence identity and assessed expression level by RNA-Seq in healthy nonsmokers. For example, in the cluster on chromosome 19 containing CYP2A6, CYP2A7 and CYP2A13 (Figure 5A), it was possible to map the reads to the different genes and show that expression of CYP2A13 (median RPKM = 17) > CYP2A6 (4) > CYP2A7 (1). In the GSTA cluster on chromosome 6 (Figure 5B), a clear assignment of reads permitted the expression order of GSTA1 (249) > GSTA2 (144) > GSTA3 (16) > GSTA5 (9). As another example, in the metallothionein gene cluster on chromosome 16 (Figure 5C), it was evident that expression level for MT1E (33) > MT1lL (2) > MT1M (1). Among the other highly expressed homologous gene families in the SAE were the α and β tubulins, annexins, glutathione S-transferase mu family, cytosolic phenol-preferring sulfotransferase family, α amylase, and NODAL modulator (Table 6). In all cases, the RNA-Seq allows the individual transcripts to be definitively distributed among family members.

**Table 6:** Different Expression Levels Among Members of Common Gene Families Expressed in the SAE^1^

Category	Gene name	Gene symbol	Median expression level (RPKM)
Cilia	tubulin, alpha	TUBA1A	341.7
		TUBA1B	162.6
		TUBA1C	120.5
	tubulin, beta	TUBB2A	38.7
		TUBB2B	23.3
		TUBB4	14.8
Annexin (signal transduction)	annexin A2	ANXA2	362.1
		ANXA2P2	292.3
		ANXA2P1	42.6
		ANXA2P3	26.1
Glutathione S-transferase alpha	glutathione S-transferase alpha	GSTA1	248.9
		GSTA2	143.9
		GSTA3	15.9
		GSTA5	9.3
Glutathione S-transferase mu	glutathione S-transferase mu	GSTM2	35.9
		GSTM1	20.8
		GSTM4	8.8
Sulfotransferase - phenol preferring	sulfotransferase family, cytosolic, 1A, phenol-preferring	SULT1A1	25.7
		SULT1A4	14.6
		SULT1A3	10.3
		SULT1A2	5.4
		SLX1A-SULT1A3	
		SLX1B-SULT1A4	
Amylase	amylase, alpha	AMY1A	43.4
		AMY1B	18.6
		AMY1C	15.1
		AMY2B	13.8
		AMY2A	7.3
Polarity/left right signaling	NODAL modulator	NOMO2	22.4
		NOMO1	16.0
		NOMO3	14.3
Metallothionein	metallothionein	MT1E	33.2
		MT1L	2.4
		MT1M	1.1
		MT1JP	
P450	cytochrome P450, family 2, subfamily A, polypeptides	CYP2A13	17.2
		CYP2A6	4.2
		CYP2A7	1.4
Aldo-keto reductase	aldo-keto reductase family 7	AKR7A2	34.1
		AKR7A3	3.0
		AKR7L	1.5
		AKR7A2P1	
Aldehyde dehydrogenase	alcohol dehydrogenase	ADH1C	52.2
		ADH1B	4.3
		ADH1A	2.6
Short chain dehydrogenase	dehydrogenase/reductase	DHRS9	68.4
		MUC20	27.5
		VPS53	4.0
		SMU1	3.9
		FAM153B	0.4
		LEP	0.0

^1 ^List of gene families identified using BLAST with ≥ 90% identity and alignment lengths of at least 50% in both sequences. They were categorized by gene family and sorted in descending order within each category by median expression level for n = 5 nonsmokers.

### Effect of Smoking on the SAE Transcriptome

**Figure 6:**
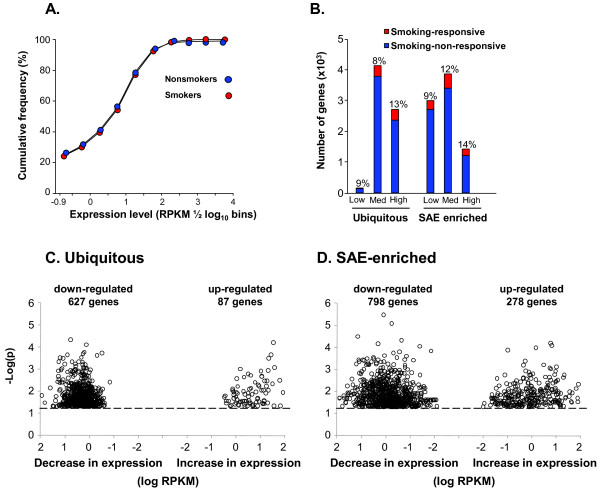
**Overall impact of smoking on small airway epithelium gene expression**. Shown are comparisons of RNA-Seq assessment of genes expressed in the small airway epithelium (SAE) in nonsmokers (n = 5) *vs *smokers (n = 6). **A**. Cumulative frequency of expression levels as a function of increasing RPKM. The data is shown as cumulative frequency in 1/2 log_10 _bins starting at the lower limit (RPKM 0.125, log_10 _= -0.9) for healthy nonsmokers (blue) and healthy smokers (red). On an overall basis assessing all genes, there is no difference in the nonsmokers *vs *smokers. **B**. Comparison of expression of the subset of smoking-responsive *vs *non-responsive genes for the ubiquitous and SAE-enriched genes. Each category is divided into low, medium and high expression groups using the same criteria as in Figures 2, 3, with smoking-responsive genes defined as p < 0.05. Ordinate - number of genes; abscissa - smoking responsive (red) and smoking non-responsive (blue) for ubiquitous and small airway epithelium (SAE)-enriched genes. Note that for both ubiquitous and SAE-enriched genes, only a small fraction, and approximately the same proportion (8-14%; low, medium, high), are smoking-responsive. **C, D**. Modified volcano plot showing absolute change in expression level (RPKM smoker - RPKM nonsmoker) *vs *-log p value for ubiquitous and SAE-enriched genes. **C**. Ubiquitous genes. **D**. SAE-enriched genes. Note that for both ubiquitous and SAE-enriched genes, more genes are down-regulated by smoking than up-regulated.

The preceding analyses of SAE-specific and ubiquitous transcripts are based exclusively on the RNA-Seq data from n = 5 nonsmokers. RNA-Seq data was also collected for n = 6 healthy smokers, who had a mean smoking history of 35 pack-yr (range of 26 to 45 pack-yr). Extensive transcription data based on the microarray methods has shown that smoking makes a substantial impact on gene expression in airway epithelium [11-18,45,46]. Quantitative comparison by RNA-Seq of SAE gene expression of healthy smokers *vs *healthy nonsmokers showed there was no gross effect of smoking on the overall distribution of the SAE transcriptome in nonsmokers and smokers (Figure 6A). However, there were changes in expression of individual genes with smoking, constituting 8 to 13% of the ubiquitous genes and 9 to 14% of the SAE-enriched genes (Figure 6B). In both categories, smoking responsiveness was slightly more noticeable in genes with medium and high expression than in genes with low expression.

To assess the quantitative effects of smoking, a modified volcano plot was devised in which the absolute change was plotted as a function of p value (Figure 6C, D). The data show that, for both the ubiquitous and for the SAE-enriched transcriptome, the number of genes down-regulated by smoking was substantially higher than that number of genes up-regulated by smoking. This was particularly noticeable among the ubiquitous genes.

**Figure 7:**
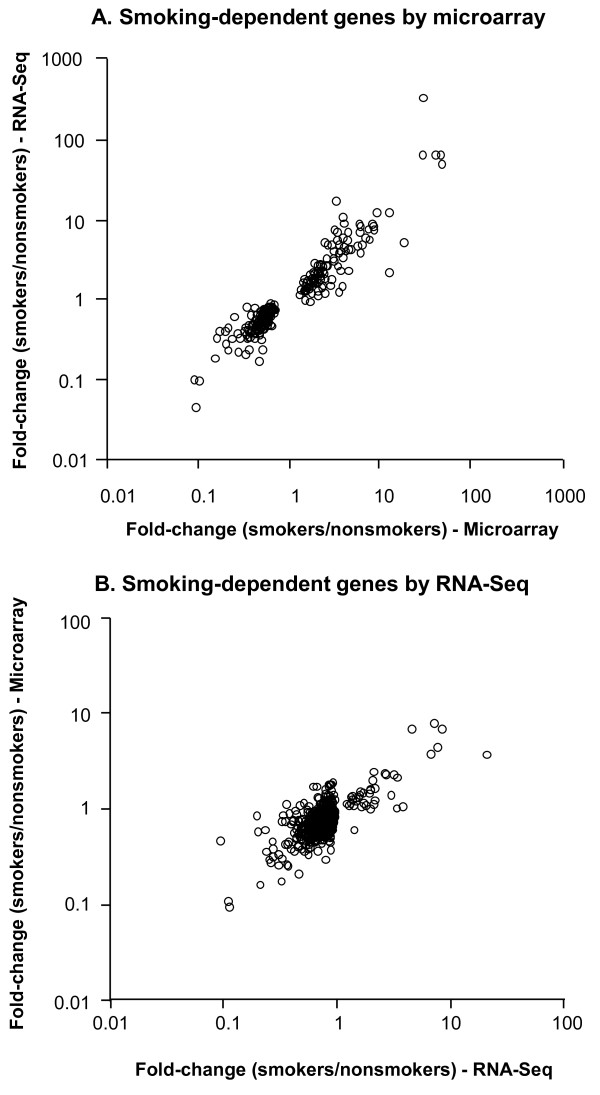
**Comparison of smoking dependent genes observed by microarray and RNA-Seq**. **A**. Microarray-determined smoking-responsive genes. The data includes all significant genes (Benjamini Hochberg corrected p value < 0.05; Additional file 1, Table S6) with > 1.5-fold different in mean expression level between n = 12 smokers and n = 12 nonsmokers, as determined by microarray. For each probeset the corresponding genes was assessed by RNA-Seq for n = 5 nonsmokers an n = 6 smokers and fold-change by microarray is plotted against the fold-change by RNA-Seq. **B**. RNA-Seq-determined smoking-responsive genes. The data includes the fold-change of all genes significantly impacted by smoking (uncorrected p < 0.005, 1.5-fold-change cut off), as assessed by RNA-Seq for n = 5 nonsmokers an n = 6 smokers. n = 12 nonsmokers and n = 12 healthy smokers were assessed by microarray and the fold-change for RNA-Seq is plotted against the fold-change for the probeset with largest change.

Because of the extensive microarray data on the response of airway epithelium to smoking, we sought to validate the RNA-Seq data by comparing the response to smoking as measured by the two different methods. Micorarray data from a cohort of 12 healthy smokers and 12 non-smokers were used to generate a list of 239 genes represented by 262 probesets that were smoking-responsive (corrected p < 0.05, no fold change cutoff) according to microarray. The fold-change by microarray was plotted against the fold change by RNA-Seq with an overall very strong correlation (r^2 ^= 0.89, Figure 7A; Additional file 1, Table S5). There were no genes for which the direction of regulation by smoking differed between microarray and RNA-Seq method. Therefore, RNA-Seq comprehensively captures the effect of smoking as determined by microarray method, thereby validating both approaches. The ability of the microarray method to capture the smoking-dependent gene expression detected by RNA-Seq was then assessed. RNA-Seq method using n = 5 nonsmokers and n = 6 smokers captured 611 smoking-dependent genes (uncorrected p < 0.005, no fold change cut off). For these genes, the impact of smoking as determined by microarray was generally similar (Figure 7B, r^2 ^= 0.58; data in Additional file 1, Table S6). While RNA-Seq faithfully captures the effects of smoking as determined by microarray, the microarray method is less discriminating in capturing the smoking-dependent expression determined by RNA-Seq, consistent with the higher sensitivity of the latter method.

**Figure 8:**
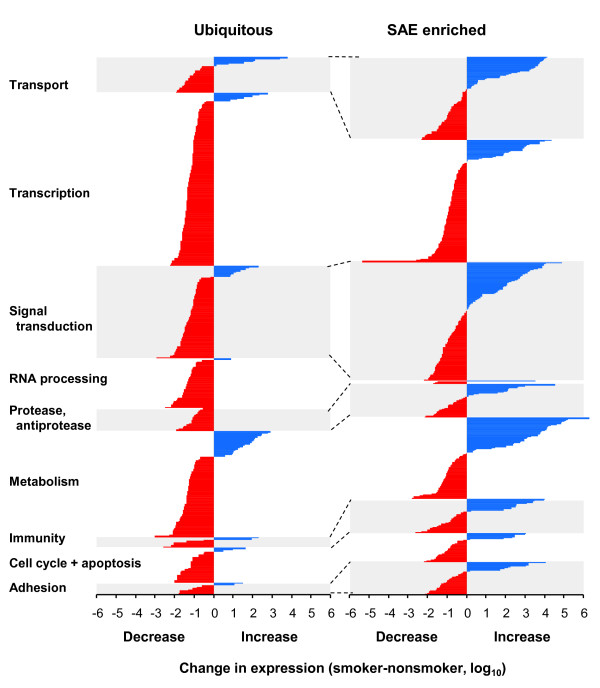
**Functional categorization of small airway epithelium (SAE) ubiquitous and SAE-enriched smoking-responsive genes**. The smoking-responsive genes (p < 0.05) of the ubiquitous and SAE-enriched groups were assigned function based on Gene Ontology classification and searches of NCBI databases. The 9 functional categories chosen accounted for the largest fraction of the genes that could be assigned functional categories. For each category, the genes were divided into smoking-induced and smoking-repressed and the log_10 _of absolute change (RPKM smoker - RPKM nonsmoker) was plotted. Genes are divided by category and ordered within each category by decreasing change in expression level. Red - down-regulated; blue - up-regulated. Note that for both ubiquitous and SAE-enriched genes, a higher fraction of genes in most categories are down-regulated, and that most up-regulated genes are in the SAE-enriched subgroup.

To assign function to smoking dependent genes, Gene Ontology searches of the Biological Process term were used to classify the expression of the smoking-suppressed and smoking-repressed genes (Figure 8). This analysis also showed that, in almost all categories, the expression of more genes was suppressed than induced. Of interest, a comparison of function in the ubiquitous and SAE-enriched categories pointed to some contrasts of potential significance. For example, among genes involved in transport, there were more smoking-induced SAE-enriched genes than ubiquitous genes and also a higher proportion of smoking-inducible genes in the SAE-enriched group compared to ubiquitous category. Similarly, among proteases and anti-proteases, there was some smoking-inducibility in the SAE-enriched genes, but none for ubiquitous genes.

In contrast to microarray data that suggests that cytochrome P450 genes and oxidant-related genes are those most highly induced by smoking [11-18,45,47], quantitative RNA-Seq analysis demonstrated the largest up-regulation of a gene by smoking was β-microseminoprotein (MSMB) and chromosome 20 open reading frame 114 (C20orf114, Table 7; see Additional file 1, Figure S1 for examples of this and other RNA-Seq-identified smoking-related genes). The most smoking-repressed genes were SCGB1A1 and SCGB3A1, which were also the two most highly expressed SAE genes in nonsmokers. The smoking-induced down-regulation of SCGB1A1 gene expression was dramatic, with an absolute decrease in median RPKM from 38,675 (13.1% of total mRNAs) to 17,244 (6.5% of mRNAs).

**Table 7:** Small Airway Epithelium Expressed Genes Most Affected by Smoking1

			RNA-Seq				Micorarray	
			
Category	Gene symbol	Gene title	Nonsmoker median	Smoker median	**Absolute difference**^**2**^	**Fold- change**^**3**^	**Fold- change**^**3**^	**p value**^**4**^
**Largest absolute increase**								
Ubiquitous	FTL	ferritin, light polypeptide	371.1	843.4	472.2	2.3	1.6	0.1784
	PRDX1	peroxiredoxin 1	187	398.6	211.6	2.1	1.6	0.1083
	FTH1	ferritin, heavy polypeptide 1	349.2	551.5	202.3	1.6	1.8	0.0602
	TUBB2C	tubulin, beta 2C	1161.3	1331.9	170.5	1.1	1.1	0.8995
	CLU	clusterin	498	665.2	167.3	1.3	1.5	0.2157
	NQO1	NAD(P)H dehydrogenase, quinone 1	38.3	198.7	160.4	5.2	4.8	< 0.0001
	UBB	ubiquitin B	615.1	711.1	96	1.2	-1.0	0.9760
	GSN	gelsolin	93.1	174.9	81.8	1.9	1.4	0.4772
	TUBA1A	tubulin, alpha 1a	341.7	423.2	81.5	1.2	1.1	0.7340
	ALDOA	aldolase A, fructose-bisphosphate	182.9	263.4	80.5	1.4	1.5	0.1670
SAE-enriched	MSMB	microseminoprotein, beta-	333.1	3112.7	2779.6	9.3	2.0	0.0820
	C20orf114	chromosome 20 open reading frame 114	1484.3	4102.7	2618.5	2.8	1.1	0.6685
	ALDH3A1	aldehyde dehydrogenase 3 family, member A1	226.9	2077.9	1851	9.2	9.8	< 0.0001
	TFF3	trefoil factor 3 (intestinal)	149.4	697.9	548.5	4.7	2.8	0.1771
	WFDC2	WAP four-disulfide core domain 2	840.3	1327	486.7	1.6	1.3	0.5735
	TPPP3	tubulin polymerization-promoting protein family member 3	1301.7	1604.9	303.2	1.2	-1.0	0.9493
	TSPAN1	tetraspanin 1	663.9	960.2	296.3	1.4	1.1	0.8884
	S100P	S100 calcium binding protein P	79.6	291.2	211.5	3.7	1.8	0.4832
	GSTA2	glutathione S-transferase alpha 2	143.9	337.5	193.6	2.3		Not in U133
	PLUNC	palate, lung and nasal epithelium associated	5.4	186	180.6	34.5	1.6	0.8375
**Largest absolute decrease**								
Ubiquitous	CRIP1	cysteine-rich protein 1 (intestinal)	1014.1	587.7	-426.3	-1.7	-1.4	0.4842
	RPLP1	ribosomal protein, large, P1	916.1	516	-400.1	-1.8	-1.2	0.3845
	CAPS	calcyphosine	2197.4	1994.7	-202.8	-1.1	-1.5	0.1733
	PRDX5	peroxiredoxin 5	1022.4	823.3	-199.1	-1.2	-1.2	0.3653
	RPS11	ribosomal protein S11	537.2	357.5	-179.7	-1.5	-1.3	0.3209
	RPLP2	ribosomal protein, large, P2	347.5	181.2	-166.4	-1.9	-1.2	0.6557
	RPL8	ribosomal protein L8	631.5	468.9	-162.6	-1.3	-1.2	0.5028
	TPT1	tumor protein, translationally-controlled 1	655.2	500.7	-154.5	-1.3	-1.1	0.6701
	S100A6	S100 calcium binding protein A6	758.3	617.2	-141	-1.2	-1.1	0.9250
	CD81	CD81 molecule	248.3	120.5	-127.8	-2.1	-1.3	0.3499
SAE-enriched	SCGB1A1	secretoglobin, family 1A, member 1 (uteroglobin)	38675.4	17244	-21431.5	-2.2	-1.1	0.4670
	SCGB3A1	secretoglobin, family 3A, member 1	7838.2	2947.3	-4890.8	-2.7	-1.3	0.1509
	CD74	CD74 molecule, major histocompatibility complex, class II invariant chain	947.2	723.7	-223.5	-1.3	-2.1	0.2881
	C9orf24	chromosome 9 open reading frame 24	628.7	488	-140.7	-1.3	-1.3	0.2256
	CYP4B1	cytochrome P450, family 4, subfamily B, polypeptide 1	373.5	259.3	-114.2	-1.4	-1.6	0.1826
	C20orf85	chromosome 20 open reading frame 85	738.1	625.3	-112.8	-1.2	-1.2	0.4756
	KRT19	keratin 19	493.4	396.8	-96.6	-1.2	-1.1	0.7722
	RPS18	ribosomal protein S18	285	207.8	-77.3	-1.4	-1.2	0.3731
	ALDH3B1	aldehyde dehydrogenase 3 family, member B1	341.9	265.1	-76.7	-1.3	-1.5	0.3595
	TMEM190	transmembrane protein 190	945.4	868.8	-76.6	-1.1	-1.2	0.5710
**Novel genes up-regulated by smoking**								
^Ubiquitous^	AHRR	aryl-hydrocarbon receptor repressor	0.1	1.3	1.2	20.8	3.7	0.0054
^SAE-enriched^	AKR1B10	aldo-keto reductase family 1, member B10 (aldose reductase)	0.3	28.5	28.2	94.8	56.6	< 0.0001
	CABYR	calcium binding tyrosine-(Y)-phosphorylation regulated	1	12.5	11.6	12.7	9.4	< 0.0001
	SPP1	secreted phosphoprotein 1	0.8	10.6	9.8	12.9	8.5	0.0021
	CYP1B1	cytochrome P450, family 1, subfamily B, polypeptide 1	0.2	9.2	9	43.3	55.0	< 0.0001
	AKR1B15	aldo-keto reductase family 1, member B15	0.1	6.5	6.3	50		Not in U133
	B3GNT6	UDP-GlcNAc:betaGal beta-1,3-N-acetylglucosaminyltransferase 6 (core 3 synthase)	0.3	4.4	4.1	16	4.5	0.0525
	NOS3	nitric oxide synthase 3 (endothelial cell)	0.6	4	3.4	6.5		"P" < 25%
	TPRXL	tetra-peptide repeat homeobox-like	0.3	3.2	2.9	10.2	3.8	0.0761
	SFRP2	secreted frizzled-related protein 2	0.3	2.7	2.4	9.8	9.7	0.0071
	FAM177B	family with sequence similarity 177, member B	0.2	2.5	2.3	11		Not in U133
**Low level expressed genes suppressed by smoking**								
Ubiquitous	PANK1	pantothenate kinase 1	1.4	0.7	-0.7	-2.1	-1.9	0.0391
SAE-enriched	LYPD2	LY6/PLAUR domain containing 2	23.9	1.7	-22.3	-14.5		Not in U133
	LYNX1	Ly6/neurotoxin 1	8.6	2	-6.6	-4.3	-1.8	0.3058
	AZU1	azurocidin 1	6.1	1.9	-4.3	-3.3	-2.1	0.2405
	ITM2A	integral membrane protein 2A	5.2	1.3	-4	-4.2	-2.6	0.0071
	ITM2A	integral membrane protein 2A	5.2	1.3	-4	-4.2	-2.6	0.0071
	SAA4	serum amyloid A4, constitutive	4.8	1.4	-3.4	-3.4	-3.4	0.0147
	GAL3ST2	galactose-3-O-sulfotransferase 2	4.7	1.6	-3.1	-2.9		"P" < 25%
	NEU4	sialidase 4	3.7	0.7	-3	-5.4	-1.2	0.6483
	PAX1	paired box 1	3.6	1	-2.6	-3.5	-1.9	0.3312
	ERP27	endoplasmic reticulum protein 27	2.4	0.5	-2	-5.2	-4.4	0.0016

^1 ^The top 20 genes were identified in four categories of genes most affected by smoking; largest absolute increase, largest absolute decrease, novel genes up-regulated by smoking and low level expressed genes suppressed by smoking. They were sorted in descending order of absolute difference in expression within each category and identified as being from the ubiquitous or small airway epithelium-enriched groups.

^2 ^Absolute difference = smoker median - nonsmoker median

^3 ^Fold change = mean in smokers/mean in non smokers

^4 ^Benjamini-Hochberg corrected p value. Where the gene is not represented on the microarray or the Affymetrix Present call "P" is less than 25% of subjects, it is so indicated.

To detect novel smoking-dependent genes, we exploited the ability of RNA-Seq to quantify expression of genes with low expression. Among the novel smoking-inducible and smoking-suppressed genes with low level expression were the smoking-inducible genes that had been previously identified by microarray (e.g., AKR1B10, CYP1B1) [11,47], but also newly identified smoking-induced genes, such as the oxido-reductase AKR1B15 and transcription factor TPRXL (Table 7). Similarly, new smoking-repressed genes were identified, including transcription factor PAX1 and AZU1, an inflammatory mediator [48].

**Figure 9:**
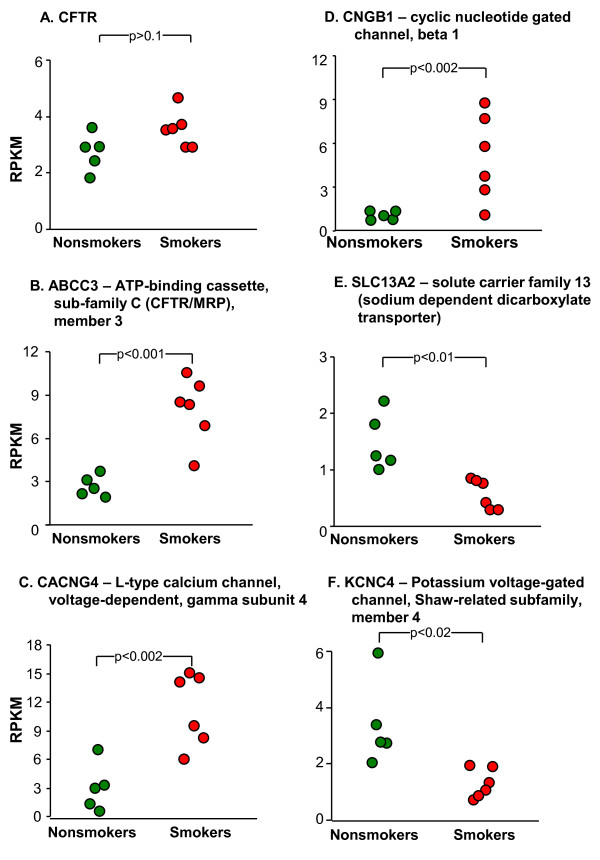
**RNA-Seq quantification of examples of smoking-dependent changes in expression of ion channel-related genes expressed in the small airway epithelium**. Smoking responsiveness of selected ion channels. **A**. CFTR - unchanged; **B-D**. Up-regulated. **B**. ABCC3; **C**. CACNG4; and **D**. CNGB1. **E-F**. Down-regulated. **E**. SLC13A2; and **F**. KCNC4. In all panels, each data point represents one individual.

Based on the number of SAE-enriched, smoking-dependent transport genes (Figure 7), we examined the expression of ion transport genes with low overall expression (Figure 8). The significance of this gene group is evident in the fact that polymorphisms in the cystic fibrosis transmembrane conductance regulator (CFTR) gene, a chloride transporter, result in cystic fibrosis, a lethal hereditary disorder with a dramatic pulmonary phenotype [49]. CFTR expression levels were in the range of 2 to 4 RPKM corresponding to an average of ~2 to 4 mRNA molecules per cell, a similar value to that estimated by polymerase chain reaction methodology [50]. There was no difference in CFTR expression level between nonsmokers and smokers (Figure 9A). In contrast, there were smoking-inducible transporters including the CFTR related ATP-binding cassette, sub-family C, member 3 (ABCC3), L-type calcium channel, voltage-dependent, gamma subunit 4 (CACNG4), and cyclic nucleotide gated channel, beta 1 (CNGB1, Figure 9B-D). There were also significantly smoking-suppressed ion transporters, including solute carrier family 13, member 2 (sodium dependent dicarboxylate transporter, SLC13A2) and potassium voltage-gated channel, Shaw-related subfamily, member 4 (KCNC4) detected (Figure 9E, F).

### Effect of Smoking on Alternative Splicing

**Figure 10:**
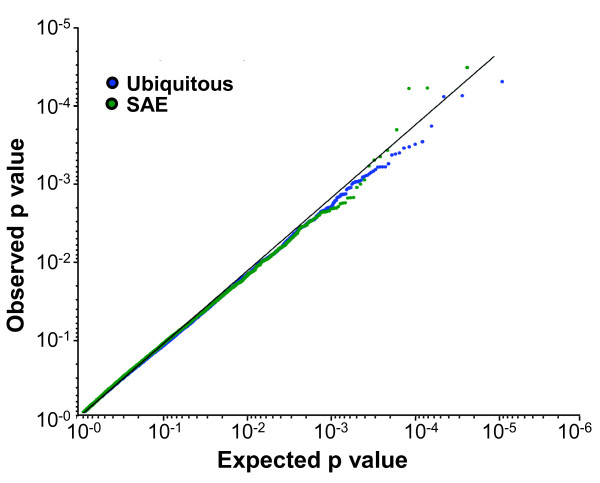
**Quantile-quantile plot of significance of difference in splice junction usage between smokers and nonsmokers**. Normalized reads supporting splicing in the smokers and nonsmoker samples were compared. The data shows that smoking caused no significant difference in the splicing for either the ubiquitously expressed genes (blue) or SAE-enriched genes (green).

Among the advantages of RNA-Seq is the ability to easily quantify different isoforms of one gene generated by alternate splicing. The frequencies of reads that span one or more splice junction were used to assess the relative levels of different isoforms separately in smokers and nonsmokers. Interestingly, comparison of the splicing events between nonsmokers and smokers to the expected distribution revealed no divergence (Figure 10). This was true for both the ubiquitous and SAE-enriched genes, suggesting there are no major smoking-dependent differences in splicing patterns.

## Discussion

The small airway epithelium, the cell population lining the bronchial tree ≥ 6 generations, plays a central role in normal lung function and in the pathogenesis of many lung disorders [7]. Among the most common SAE-associated diseases are those caused by cigarette smoking, including chronic obstructive pulmonary disease (COPD) and lung adenocarcinoma. The development of massive parallel RNA sequencing (RNA-Seq) technology permits quantitative assessment of poly(A)^+ ^mRNA levels to a high degree of sensitivity [19-24]. Compared to hybridization-based methodologies of transcriptome analysis, RNA-Seq has low background, broad dynamic range and high specificity [22]. Using this approach, we have built upon the body of microarray-generated data to provide quantitative characterization of the transcriptome of the normal healthy human SAE and characterize how it changes with smoking [11-18]. By comparing the SAE RNA-Seq data to that of other tissues and organs, the present study grouped the SAE transcriptome into 2 categories: (1) ubiquitous genes, i.e., SAE genes shared with other organs and tissues, and (2) SAE-enriched genes, i.e., those expressed by the SAE, but not in the majority of other organs and tissues. Using this classification, and based on the capacity of RNA-Seq to provide quantification of mRNA, we further characterized the effect of smoking on the SAE transcriptome.

### SAE-enriched Transcripts

Comparison of the expression profile of different tissues by RNA-Seq and Serial Analysis of Gene Expression (SAGE) allows the identification of ubiquitous and tissue specific genes [24,51]. By comparing to the RNA-Seq data obtained for other organs and tissues, we found that among 15,877 genes expressed in the SAE, 52% of genes are enriched in the SAE in a relatively selective manner and 48% of genes are ubiquitous. Interestingly, the SAE transcriptome includes more tissue-characteristic RNAs than other epithelial (breast, kidney, colon) and non-epithelial (heart, brain, skeletal muscle, adipose tissue, lymph node) tissues, where ubiquitous genes contribute to 65 to 85% of the transcriptome [24]. This may reflect the high purity of the epithelial cells obtained by bronchial brushing (i.e. they are not contaminated by endothelium, connective tissue or inflammatory cells and therefore do not appear to express genes that are expressed by contaminating cell types). Notably, SAE genes with low expression levels contributed to 36% of the SAE-enriched and only 2% of ubiquitous genes, indicating that molecular uniqueness of the SAE is determined to a considerable degree by the transcripts with a low abundance. From the functional perspective, ubiquitous SAE genes dominated in the categories related to housekeeping biologic processes such as translation and transcription, whereas SAE-enriched genes were prevalent in more specific categories such as immunity, signal transduction, adhesion, and ion transport.

### SAE Transcriptome Specialization

Specialized biological properties of a given organ or tissue are determined by a unique pattern of genes expressed in distinct cell populations typical for each tissue. The human SAE is composed of various cell types, including ciliated, secretory (mostly Clara cells but also surface epithelium mucus-producing cells), basal, undifferentiated columnar, and rare neuroendocrine cells [1,2,13,31,52]. Although most of the SAE-enriched genes are represented by low expressed transcripts, the top 30 highly expressed SAE-enriched genes accounted for about 20% of the total SAE mRNA, suggesting that a limited number of genes may dictate the specific pattern of biological processes dominating in the SAE under steady-state conditions. Detailed analysis of the most highly expressed SAE-enriched genes revealed a unique pattern of epithelial differentiation and molecular functions.

#### Secretory differentiation

Genes related to secretory epithelial differentiation dominated the most highly expressed SAE-enriched genes. Of the total SAE transcripts identified, 13% mapped to the secretoglobin SCGB1A1. The high level of expression of SCGB1A1 is expected in the SAE, where Clara cells are enriched and play an important role in the pulmonary host defense [27-29]. SCGB1A1, is involved in regulation of critical processes in the distal airways such as protection against oxidative stress, maintenance of the normal airway lining fluid homeostasis, regulation of inflammation and airway reactivity during respiratory infection, and control of macrophage activation in the lung [53-57]. Another secretoglobin, SCGB3A1, originally called HIN-1, was the second-highest expressed SAE-enriched gene. Previous studies have identified the lungs as major site of SCGB3A1 in humans [58]. Expression of SCGB3A1 is induced during epithelial differentiation and restricted to terminally differentiated airway epithelial cells and down-regulated in cancer [58,59]. There is evidence that SCGB3A1 is also produced by Clara cells [60] and exerts growth-inhibitory activities [61]. Consistent with its putative tumor-suppressor function, SCGB3A1, is aberrantly methylated in various types of cancer, including lung carcinomas [62]. Based on previous observations, the quantitative data in the present study suggests that SCGB3A1 could be a major steady-state tumor-suppressor gene in the human SAE.

High expression of Clara cell-associated secretoglobin genes in the SAE was accompanied with enrichment of transcription factors forkhead box A1 (FOXA1), NK2 homeobox 1 (NKX2-1), FOXA2, and CCAAT/enhancer binding protein, alpha (C/EBPα), transcription factors that constitute a major regulatory network for the development and maintenance of SAE and Clara cell differentiation [43,63-65]. NKX2-1 interacts with FOXA1 [34], FOXA1 and FOXA2 complement each other [35], and both NKX2-1 and FOXA2 are thought to act upstream of C/EBPα in lung epithelial differentiation [65,66]. A number of secretory genes, not previously described for the human SAE, were identified by RNA-Seq as highly abundant SAE-enriched genes, including tetraspanin-1 (TSPAN1), a protein involved in secretory pathways in glandular cells [67], cytochrome CYP4B1, a CYP family member localized within the secretory granules of the respiratory mucosa [68], and microseminoprotein-beta (MSMB), an androgen-responsive secretory protein regulating cell growth and apoptosis [69].

#### Mucosal host defense

Secretory leukocyte peptidase inhibitor (SLPI) and polymeric immunoglobulin receptor (PIGR), two key components of the mucosal defense system, were among the most highly expressed SAE-enriched genes. SLPI has multiple contributions to pulmonary defense, including its ability to neutralize neutrophil elastase, one of the major mediators of lung derangement associated with inflammatory diseases, direct antimicrobial and anti-inflammatory activities, and augmentation of anti-oxidant resistance by increasing glutathione levels in the respiratory surface fluid [70-73]. PIGR is essential for the transepithelial basal-to-apical transport of the polymeric immunoglobulin IgA to the epithelial surface, where it functions to sample and neutralize luminal pathogens [74]. Lipocalin 2 (LCN2), a siderophore-binding antimicrobial protein secreted by pulmonary epithelial cells [75], and the whey acid protein four-disulfide core domain 2 (WFDC2), a SLPI-related gene with potential innate immune functions [76], were also among the most abundant genes enriched in the SAE. Among the most highly expressed genes in the SAE was ELF3, a helix-loop-helix transcription factor expressed in diverse epithelial tissues implicated in the regulation of inflammatory responses [39]. In the context that the airway epithelium is at the interface of the environment (the apical surface) and potential inflammatory/immune mediators (the basolateral surface), the host defense genes identified in the present study as the most abundant SAE genes may play a central role in both mediating and controlling the responses of the airway to environmental xenobiotics and pathogens.

#### Anti-oxidant protection

The ability to resist deleterious effects of the oxidative stress is critical for the SAE, continuously interacting with oxidants present in the inhaled air. Apart from the secretory genes with anti-oxidant functions such as SCGB1A1 and SLPI, a number of other genes directly related to the protection from oxidative stress, including glutathione S-transferases pi 1 and alpha 1, and glutathione peroxidase 1 (GPX1), were identified as highly expressed SAE-enriched genes. One of these components, GPX1, also known as Clara cell-specific protein CC26, is selectively expressed by Clara cells [55], suggesting that high abundance of both secretory and oxidative stress-related features in the SAE might reflect a secretory cell origin of at least some of the anti-oxidant mechanisms in the human SAE.

#### Mucociliary differentiation

Consistent with the abundance of ciliated cells in the human SAE, transcription factor FOXJ1, the major regulator of ciliogenesis and ciliated cell differentiation in the airway epithelium [41,42], was among the top 20 SAE-enriched genes and the most highly expressed transcription factor. Other cilia-related genes enriched in the SAE were tektin-1 and -2, structural determinants of the basal bodies of cilia [77], cilia apical structure protein sentan [78], dynein chains DNAI1, DNALI1, DNAI2 and sperm associated antigen SPAG6, the classic components of motile cilia [79]. In addition to these well-known genes, RNA-Seq analysis revealed that several recently discovered cilia-related genes were highly enriched in the human SAE, including the member of the membrane-spanning 4-domain family MS4A8B, which has high sequence homology to cilia-associated gene L985P [80].

Surprisingly, a considerable number of mucus-related genes, such as trefoil factor 3 [81], mucin 1 and mucin 5B [82,83], were highly expressed in the SAE transcriptome along with AGR2, a secretory factor related to goblet cell differentiation [84,85]. Of note, as compared to the large airways, where secreted polymeric mucins are abundant [86], the SAE transcriptome was enriched in membrane-tethered mucins such as MUC1, MUC4, MUC15, MUC20, MUC16, and MUC13, which have various signaling functions [87].

#### Stem/progenitor cell features

Although Clara cells are considered to be stem/progenitor cells of the mouse bronchiolar epithelium [8,88], the identity of stem/progenitor cell population of the SAE in humans is unknown. Several genes related to stem/progenitor cells were identified in the present study as SAE-enriched genes, including aquaporin-3, a marker of basal cell and suprabasal cell populations with progenitor cell function described for the human tracheobronchial epithelium [89] and aldehyde dehydrogenase ALDH1, a marker of normal and malignant stem cells in various tissues [90,91]. It is notable that among the top 5 highly-expressed SAE-enriched transcription factors were ELF3, which promotes epithelial morphogenesis [92], and embryonic stem cell-related gene SOX2, recently shown to be important for the progenitor cell function of the airway basal cells and Clara cells and induction of the airway epithelial cell phenotype in mice [36-38]. Due to its high sensitivity, RNA-Seq analysis also identified markers of the putative stem/progenitor cells previously found in the airway epithelium with relatively low frequency, such as keratin 14, a marker of a basal cell subpopulation [93], and surfactant protein C, a gene ascribed to a unique population of bronchoalveolar stem cells in mice [88]. Together, the RNA-Seq data of the present study demonstrates SAE expression of multiple pathways potentially relevant for the maintenance of hu-man SAE via local stem/progenitor cell activity.

#### Transmembrane receptors, signaling ligands and growth factors

The most highly expressed transmembrane receptor in SAE of nonsmokers was DDR1 (discoidin domain receptor 1), a receptor tyrosine kinase [44]. Expression of the DDR1 protein is located on the basolateral surface of human bronchial epithelium, where it interacts with type IV collagen with consequent activation of its tyrosine kinase activity. The second most abundant SAE-enriched receptor was CELSR1 (cadherin, EGF LAG seven-pass G-type receptor 1), a G protein coupled receptor known to be critical for branching morphogenesis in mouse lung [94]. The most highly expressed SAE-enriched ligand was midkine (MDK), which has a role in lung morphogenesis and is believed to be essential for vascular maintenance in the adult lung [95]. In mouse, midkine expression is controlled by Nkx2-1 [96] which, as mentioned above, is also highly expressed in the human SAE. Among the highly expressed ligands, there was a clear prevalence of chemokines such as MDK, CXCL1 and CX3CL1. Consistent with this observation, expression of diverse cytokines by airway epithelium and cell lines derived from airway epithelium is well established and epithelial derived chemokines are recognized to play an important role in attracting immune and inflammatory cells [97,98].

The RNA-Seq data also pointed to potentially novel aspects of cell signaling in epithelial biology. For example, the oxytocin receptor (OXTR) was expressed at high levels in all subjects who were male. This was initially surprising due to roles of oxytocin in childbirth, lactation and brain biology [99] but, relevant to the airway epithelium, a role for oxytocin in autocrine signaling in small cell lung cancer has been described [100].

### Gene Family Members

RNA-Seq offers the potential advantage of distinguishing expression levels among different members of closely related gene families with potentially different functions, whereas cross hybridization among probes often makes this a challenge using microarrays [25]. For example, the RNA-Seq analysis permitted quantification of the expression levels of 3 highly different homologous members of the cytochrome P450 family 2, subfamily A, CYP2A6, CYP2A7 and CYP2A13. RNA-Seq allowed the transcripts to be unambiguously attributed primarily to CYP2A13 which is responsible for metabolism of the cigarette smoke specific carcinogen 4-(methylnitrosamino)-1-(3-pyridyl)-1-butanone [101]. On the other hand, the family member CYP2A6 has different substrate specificity dictated by critical amino acid differences between these otherwise closely related proteins [102]. The significance of these differences are underscored by the variant CYP2A13*2, which is associated with decreased incidences of lung adenocarcinoma in smokers [103].

### SAE Transcriptome Response to Smoking

Extensive microarray studies have identified a dramatic effect of smoking on the gene expression profile of human airway epithelium [11-18]. By using RPKM quantification as a measure of smoking-dependent changes in SAE transcript levels, the present study expands the insights into the airway epithelial response to smoking. The quantitative RNA-Seq analysis revealed that smoking suppressed the expression level of a greater number of genes than it induced. Interestingly, among the up-regulated genes, smoking has a larger effect on SAE-enriched genes rather than the ubiquitous genes. From the functional perspective, the SAE-enriched smoking-up-regulated genes were related to transcription, signal transduction, protease/antiprotease homeostasis, and immunity.

The top 2 SAE-enriched genes, the Clara cell associated genes SCGB1A1 and SCGB3A1, were both down-regulated by smoking with a large magnitude of change in expression levels. Smoking and especially COPD have been associated with the loss of Clara cells and the levels of SCGB1A1 in both induced sputum and serum are lower in smokers with COPD as compared to both nonsmokers and healthy smokers [104-107]. It is possible that down-regulation of Clara cell secretoglobins, with their anti-oxidant, anti-inflammatory and tumor-suppressor activities [60,108,109], is a critical component of smoking-related development of COPD. The decreased number of SCGB1A1-expressing Clara cells in smokers is generally accompanied by an increased frequency of mucin-secreting cells [29]. Indeed, a subset of highly expressed SAE-enriched genes, such as C20orf114 (also known as long PLUNC1), and MSMB, both associated with mucin-producing secretory cell phenotype [110,111], were among the smoking-induced genes with the highest amplitude of up-regulation. Other genes related to a secretory phenotype such as WFDC2, TSPAN1, TFF3, S100P, and short PLUNC, were also induced by smoking; each of these genes has been associated with epithelial carcinogenesis [67,112-115]. Thus, a broad induction of a mucin-producing cell secretory program, characteristic of epithelial malignancies, may represent an early molecular phenotype relevant to smoking-induced carcinogenesis in the distal airways.

Other smoking-induced changes among the highly expressed SAE-enriched genes included up-regulation of oxidative stress-related genes ALDH3A1 and GSTA2, also associated with cancer development [116,117], and down-regulation of genes associated with epithelial differentiation such as CD74, C9orf24 (also known as ciliated bronchial epithelium 1), and luminal cell-associated keratin 19[118,119]. Some of these changes have not been previously detected by microarrays, likely due to microarray saturation of signal with high levels of expression and/or higher sensitivity of the RNA-Seq methodology to gene expression changes with relatively low overall fold-difference between the groups.

The ability of RNA-Seq to assess genes with low steady-state expression was utilized in the present study to characterize the effect of smoking on the expression of low abundance SAE genes. Although some of changes, such as up-regulation of oxidative stress-responsive genes AKR1B10, CABYR, and CYP1B1 have been previously reported [11,45-47], RNA-Seq quantification revealed a number of novel smoking-responsive genes, including smoking-induced NOS3, a gene encoding nitric oxide isoform usually expressed by endothelial cells but induced in the airway epithelium in association with squamous differentiation [120], and smoking-suppressed Ly6/neurotoxin 1 (LYNX1), an allosteric modulator of nicotinic acetylcholine receptors [121].

Functional classification of the low level, smoking-related genes also identified the class of ion transport genes as being modulated by smoking. One example was CNGB1, a smoking-induced gene that encodes a cyclic nucleotide gated channel that was first identified for its role in light activated cellular polarization in retinal photoreceptor cells [122] and linked to olfactory receptor function [123]. The discovery that airway ciliated cells have olfactory receptors that operate by the same signal transduction pathways as visual rhodopsin [124] suggests a role for CNGB1 in airway epithelial function. Also notable among smoking-dependent genes were a series of ion channels whose overall low expression level in the SAE may reflect expression predominantly in neuroendocrine cells which constitute < 0.01% of total airway epithelium. For example, CACNG4, the gamma subunit of a voltage gated calcium channel, is a smoking-induced gene. Previous reports suggest that this gamma subunit is expressed primarily in brain [125] but expression of voltage gated calcium channels in neuroendocrine cells and neuroendocrine-derived tumors has been demonstrated [126].

### Splicing

The mRNA sequence reads across exon junctions permit quantitative assessment of the splicing pattern for all genes. By comparing splice events for smokers and nonsmokers, we were able to demonstrate there are no overall smoking-dependent changes in the patterns of splicing for either ubiquitous or SAE-enriched genes. This was surprising, since there are known to be substantial genome wide differences in splicing between normal airway epithelium and lung cancer [127,128], suggesting those splicing-related changes are late events and are not represented in non-transformed airway epithelial cells.

## Conclusions

RNA-Seq method provides wide dynamic range and low noise. Application of RNA-Seq to SAE allowed the unequivocal identification of highly expressed ubiquitous and SAE-enriched genes. Functional assignment of highly expressed genes showed Clara cell specific genes were most abundantly expressed. But genes characteristic of minor cell types such as neuroendocrine cells were also evident. Comparison of the transcriptome of nonsmokers to that of healthy smokers allowed the response of SAE to cigarette smoke to be quantified and novel smoking-responsive genes to be identified.

## Methods

### Study Population

Following approval by the Weill Cornell Medical College Institutional Review Board, healthy nonsmokers and healthy smokers, who responded to local advertisements regarding a research study to assess lung health, were assessed in the Weill Cornell National Institutes of Health Clinical and Translational Sciences Center and Department of Genetic Medicine Clinical Research Facility. Prior to study enrollment, each individual provided written informed consent. The study population included healthy nonsmokers (n = 5) and healthy smokers (n = 6), phenotyped by a standardized screening assessment consisting of a history, physical examination, complete blood count, coagulation profile, liver function tests, urine studies, chest X-ray, EKG, and lung function tests (see Additional Data Methods for inclusion/exclusion criteria; Additional file 1, Table S1 for detailed demographics). Urinary nicotine and cotinine were used to verify the self-reported smoking status of smokers. For comparison between RNA-Seq and microarray data, 27 healthy nonsmokers from a previous study were assessed [129] (see Additional file 1, Table S1 for demographic details).

### Collection of SAE

Fiberoptic bronchoscopy was used to sample SAE cells as previously described [13]. After routine anesthesia, a 2 mm disposable brush (Wiltek Medical, Winston-Salem, NC) was inserted into the working channel of the bronchoscope and advanced to the airways distal to the orifice of the desired lobar bronchus. Small airway epithelial samples were obtained by lightly wedging the brush 7 to 10 cm distal to the 3^rd ^generation bronchial airway (i.e., the 10^th ^to 12^th ^order bronchi), and sliding the brush back and forth on the epithelium 10 to 20 times in 8 to 10 sites. For each brush, after withdrawing from the bronchoscope, the cells were dislodged from the brush by flicking the brush tip in 5 ml of ice-cold Bronchial Epithelium Basal Medium (BEBM, Lonza, Basel, Switzerland). A 1 ml aliquot of all airway epithelial samples was used to quantify the percentage of epithelial and inflammatory cells and the proportions of basal, ciliated, secretory and undifferentiated columnar cells by centrifuging 2 × 10^4 ^cells per slide (Cytospin 11, Shandon Instruments, Pittsburgh, PA) and using Diffquik staining reagents (Dade Behring, Newark, NJ); a portion of this aliquot was also used to quantify of the number of cells recovered from airway brushings using a hemocytometer. The remaining 4 ml of sample was im-mediately processed for RNA extraction.

### RNA Extraction and Sample Preparation

The freshly acquired small airway epithelial samples were treated with TRIzol (Invitrogen Carlsbad, CA) to extract total RNA, and residual DNA was removed by RNeasy MinElute RNA purification kit (Qiagen, Valencia, CA), resulting in a yield of between 2 and 4 :g RNA per 10^6 ^cells. To assess the integrity of the RNA, an aliquot of each sample of RNA was analyzed with the Agilent Bioanalyzer (Santa Clara, CA), and the NanoDrop ND-1000 spectrophotometer (NanoDrop Technologies, Wilmington, DE) was used to determine the RNA concentration. Samples were then stored in RNAsecure (Ambion, Austin, TX) until further analysis. Using the reagents of the mRNA Sample Prep Kit and in accordance with the RNA sequencing protocol provided by Illumina, poly(A)^+ ^mRNA was selected out from the total RNA samples using Sera-mag magnetic oligo(dT) beads. An RNA fragmentation kit (Ambion, Austin, TX) was used to fragment the mRNA, followed by first- and second-strand cDNA synthesis using random hexamer primers. An "end repair" reaction to blunt the ends of all fragments was then performed with Klenow polymerase and T4 DNA polymerase, and 3'- to 5' exo-nuclease was used to create a 3' adenine overhanging tail, facilitating the ligation of the amplification adapters. Ligation products were then separated on a 2% tris-acetate-EDTA-agarose gel for size selection, followed by purification with a gel extraction kit. The purified ligation products were then PCR amplified with complementary primers and the resultant cDNA was purified with QIAquick PCR kit (Qiagen), and the concentration was measured by the NanoDrop spectrophotometer. Samples were then loaded onto Illumina flow cells for single end, 43 nucleotide, sequencing reactions.

### Data Filtering, Read Mapping and Quantification of Gene Expression

Images acquired by the Illumina Genome Analyzer 2 were analyzed by Firecrest and bases called by Bustard (both part of Illumina RTA pipeline version 1.6). All lanes of data were required to show low overall error rate (< 1.5%), low inter-base phasing (< 1.0), and all reads passed the Illumina GA quality filters (PF = Y). Resultant reads were aligned to the reference genome build UCSC hg19 using Bowtie v 0.12 [130]. The Bowtie default parameters used were those in which the first seed alignment of size > 28 nt, allowing up to 2 mismatches, is chosen at random, and is used if it yields an alignment quality sum of > 70 with a maximum of 125 backtraces. Thus, multiple alignments are not specifically assessed nor scored. The data was then processed with Python scripts to assign aligned reads to the coordinates of exons and genes. Mean read density values for exons, introns, and intergenic regions were computed in units of reads per kilobase of exon/intron/intergenic per million mapped reads [23]. Reads were mapped to the annotated transcribed strand of the genome, because the protocol for sequencing used in the current study was not strand specific. Reads per kilobase of exon per million mapped reads (RPKM) are indicative of actual mRNA concentration when samples have relatively uniform sequencing coverage across the entire gene model [23].

To determine the minimum detectable level of expression, a false discovery rate (FDR) and false negative rate (FNR) was estimated by comparing the expression levels of known exons to intergenic regions (Figure 1). This was done in accordance with the method described by Ramsköld et al. [24]. The distribution of exon expression levels was compared to the expression levels of intergenic regions based on the criteria: (1) no annotated genes according to the NCBI Reference Sequence (RefSeq; http://www.ncbi.nlm.nih.gov/RefSeq/) and Ensembl http://www.ensembl.org databases; and (2) no known expressed sequence tags in the GenBank sequence database http://www.ncbi.nlm.nih.gov/genbank. In order to avoid a bias due to changes in the size distribution of intergenic regions and exons, the intergenic regions were chosen at random to have the same size distribution of the expressed exons. The FDR was calculated for different expression levels as the normalized ratio of number of intergenic regions to number of exons at each expression level. The FNR for different expression levels was estimated from the cumulative ratio of the true positive rate (as estimated from the product of number of expressed exons and the FDR) and the total fraction of expressed exons. Based on this analysis, the optimal expression value as defined by the intersection of the FDR and FNR in all non-smoker samples was 0.125 RPKM.

All sequence read data have been submitted to the Short Read Archive (SRA) section of the NCBI SRA database (SRA accession #SRP005411); and U133 data submitted to GEO (GSE27681).

### Data Analysis

A cut off value of RPKM 0.125 was established, below which expression was considered as noise (Figure 1). Genes for which the median expression in nonsmokers was > 0.125 RPKM were scored as expressed. Genes expressed by the small airway epithelium (SAE) were categorized as "ubiquitous" and "SAE-enriched" as follows. Ubiquitous genes were defined as described by Ramsköld et al. [24] based on expression in 11 of 12 tissues surveyed. The SAE expressed genes were grouped as "ubiquitous" if also expressed in at least 11 of 12 other tissues or "SAE-enriched" (if not in the "ubiquitous" list) [24]. Based on the median expression level in nonsmokers, expressed genes were further divided into "low" (RPKM 0.125 to 1), "medium" (RPKM between 1 and 10) and "high" expression genes (RPKM > 10).

The abundance of the transcripts from individual genes in the total mRNA pool in different tissues was assessed by building a frequency distribution. For our SAE data and for published RNA-Seq data from various tissue [24], all genes were ranked by transcript abundance and then the fraction of total mRNA contributed by gene #1, genes #2-10, genes #11-100, genes #101-1000 and # > 1000 was determined. This gives a frequency distribution that could be compared among tissues with assessment by Fisher's exact test.

To compare the RNA-Seq data to that of microarrays, Human Genome U133 Plus 2.0 microarray data (Affymetrix, Santa Clara, CA) from 27 healthy, African-American nonsmokers was used ([129]; Additional file 1, Table S1). The microarray CEL files were analyzed by Affymetrix Suite software and the "P" calls for each probeset were totaled for all subjects. The gene list from RNA-Seq was systematically reviewed in comparison to the microarray data. Where there was a corresponding probeset on the microarray data, the percentage of subjects with "P" call was determined. When there was > 1 probeset corresponding to a single named gene, the probeset with the highest percentage P call was used.

To further characterize the healthy SAE transcriptome, the data from the healthy nonsmokers were assessed for: (1) the overall most highly expressed genes; (2) the most highly expressed genes of differentiated cell types (ciliated, secretory, basal and neuroendocrine cells), using lists of genes characteristic of these differentiated cell types (Additional file 1, Table S4) [30,31,52]; (3) genes coding for transcription factors; (4) genes coding for transmembrane receptors; and (5) genes coding for signaling ligands and growth factors. In all cases, the most highly expressed was based on the median for all nonsmokers. Gene families expressed by the SAE of healthy nonsmokers were identified using Basic Local Alignment Search Tool (BLAST; http://www.ncbi.nlm.nih.gov/BLAST/). All RefSeq genes expressed by nonsmokers were aligned against a database of all human RefSeq mRNA [26]. Gene families were defined as groups of genes for which the alignments yielded ≥ 90% identity and the alignment length was at least 50% of both the query and matched sequences. Changes in gene expression of the family members were assessed as described above.

Smoking responsive genes were assigned on the basis of comparing RPKM level in 5 nonsmokers to that in 6 smokers by t-test with no correction for multiple comparisons. All genes with a p value of < 0.05 were deemed to be smoking-dependent regardless of any cut off in absolute change or fold-change (smoker/nonsmoker expression ratio).

The effect of smoking on alternative splicing was estimated by comparing normalized splice junction usage. To accomplish this, all reads that failed to align to the reference genome were aligned (using Bowtie) to a database of all RefSeq annotated exon-exon boundaries generated such that each junction required reads to overlap each exon by at least 3 nucleotides. By normalizing the number of reads at each junction by the length of each junction in kilobases, number of reads in the sample in millions of reads and the expression level of neighboring exons, it was possible to compare junction usage even in genes with different expression levels. To re-duce the false positive rate, filtering included exclusion of all junctions with expression levels (RPKM) below 0.125, all junctions with less than 2 spliced reads in both the smoker and non-smoker samples, as well as any genes where the standard error in RPKM across all samples was greater than 0.5. A t-test was used to estimate the significance of the difference in splice junction usage of the filtered junctions between smokers and nonsmokers. The data were analyzed using multiple test corrections with evaluation by Q-Q plot.

## List of abbreviations

RNA-Seq: high throughput sequencing of mRNA fragments; SAE: small airway epithelium; COPD: chronic obstructive pulmonary disease; FDR: false discovery rate; FNR false negative rate; cDNA: complementary DNA; RPKM: reads per kilobase of exon per million mapped reads.

## Competing interests

The authors declare that they have no competing interests.

## Authors' contributions

NRH, MWB and RS analyzed data, wrote article, mined data for biological meaning; JS, JLRF, JGM, YS-B performed bioinformatic and statistical analyses; LO, analyzed splicing; GW analyzed and interpreted data related to secretory cells; LD analyzed and interpreted data related to transcription factors; RGC conceived and guided the overall project. All authors have read and approved the final manuscript.

## Supplementary Material

Additional file 1 **Additional Data Methods**. Additional Table S1. Demographics of the study population and biologic samples. Additional Table S2. Mapping summary. Additional Table S3. Comparison of the median expression levels of different categories of genes in the small airway epithelium of healthy nonsmokers and healthy smokers. Additional Table S4. Cell type-specific gene lists. Additional Table S5. Reproducibility of smoking-responsive genes discovered by microarray using RNA-Seq method. Additional Table S6. Reproducibility of smoking-responsive genes discovered by RNA-Seq using microarray method. Additional Figure Legends. Additional figures S1 and S2. Click here for file
