# Supplementary material for: RNA-Seq quantification of the human small airway epithelium transcriptome
Source: BMC Genomics. 2012 Feb 29;13:82. doi: 10.1186/1471-2164-13-82 (PMC3337229; doi:10.1186/1471-2164-13-82)
Supplement: Additional file 1 — Additional Data Methods. Additional Table S1. Demographics of the study population and biologic samples. Additional Table S2. Mapping summary. Additional Table S3. Comparison of the median expression levels of different categories of genes in the small airway epithelium of healthy nonsmokers and healthy smokers. Additional Table S4. Cell type-specific gene lists. Additional Table S5. Reproducibility of smoking-responsive genes discovered by microarray using RNA-Seq method. Additional Table S6. Reproducibility of smoking-responsive genes discovered by RNA-Seq using microarray method. Additional Figure Legends. Additional figures S1 and S2. [file 1471-2164-13-82-S1.PDF]

Additional Data Methods

Healthy Nonsmokers and Healthy Smoker Inclusion and Exclusion Criteria

Healthy nonsmokers

Inclusion criteria

- Males and females, at least 18 years old
- Provide informed consent
- Good health without history of chronic lung disease, including asthma, and without recurrent or recent (within 3 months) acute pulmonary disease
- Normal physical examination
- Normal routine laboratory evaluation, including general hematologic studies, general serologic/immunologic studies, general biochemical analyses, and urine analysis
- HIV-1 negative, normal α1-antitrypsin level
- Normal PA and lateral chest X-ray
- Normal electrocardiogram (sinus bradycardia, premature atrial contractions are permissible)
- Not pregnant (females)
- No history of allergies to medications used in the bronchoscopy procedure
- Not taking any medications relevant to lung disease or having an effect on the airway epithelium
- Willingness to participate in the study
- Self-reported nonsmokers, with smoking status validated by the absence of nicotine and cotinine in urine

Exclusion criteria

- Unable to meet the inclusion criteria
- Current active infection or acute illness of any kind
- Alcohol or drug abuse within the past 6 months
- Evidence of malignancy within the past 5 yr

Healthy active smokers

Inclusion criteria

- Males and females, at least 18 years old
- Provide informed consent
- Good health without history of chronic lung disease, including asthma, and without recurrent or recent (within 3 months) acute pulmonary disease
- Normal physical examination
- Normal routine laboratory evaluation, including general hematologic studies, general serologic/immunologic studies, general biochemical analyses, and urine analysis
- HIV-1 negative, normal α1-antitrypsin level
- Normal PA and lateral chest X-ray
- Normal electrocardiogram (sinus bradycardia, premature atrial contractions are permissible)
- Not pregnant (females)
- No history of allergies to medications used in the bronchoscopy procedure

- Not taking any medications relevant to lung disease or having an effect on the airway epithelium
- Willingness to participate in the study
- Self-reported current daily smokers with any number of pack-yr, validated by urine nicotine >2 ng/ml and/or cotinine >5 ng/ml

Exclusion criteria

- Unable to meet the inclusion criteria
- Current active infection or acute illness of any kind
- Alcohol or drug abuse within the past 6 months
- Evidence of malignancy within the past 5 yr

Additional Table S1. Demographics of the Study Population and Biologic Samples[1]

Parameter	RNA-Seq[2] Healthy nonsmokers	RNA-Seq[2] Healthy smokers	Microarray[2] Healthy nonsmokers
n	5	6	27
Sex (male/female)	5/0	6/0	18/9
Age (yr)	36.0 ± 10.8	49.0 ± 3.8	37.3 ± 9.8
Race (B/W/O)[3]	5/0/0	6/0/0	27/0/0
Smoking history (pack-yr)	-	35.2 ± 7.5	-
Urine nicotine (ng/ml)	-	1359 ± 887	-
Urine cotinine (ng/ml)	-	1601 ± 952	-
Pulmonary function parameters[4]			
FVC	105 ± 17	116 ± 12	105 ± 13
FEV1	106 ± 11	115 ± 16	107 ± 12
FEV1/FVC	82 ± 10	78 ± 7	84 ± 7
TLC	90 ± 4	100 ± 16	96 ± 10
DLCO	103 ± 30	88 ± 6	96 ± 15
Total cells[5]			
Number recovered $x10^6$	7.1 ± 2.0	10.1 ± 4.5	5.7 ± 1.8
% epithelial cells	99.3 ± 1.0	99.7 ± 0.3	99.2 ± 0.9
% inflammatory cells	0.7 ± 1.0	0.3 ± 0.3	0.8 ± 1.0
Differential cell count[6]			
Ciliated (%)	72.7 ± 5.5	62.6 ± 6.1	73.0 ± 10.2
Secretory (%)	7.3 ± 6.1	9.3 ± 4.2	6.8 ± 4.1
Basal (%)	10.4 ± 6.4	11.9 ± 2.5	12.6 ± 8.0
Undifferentiated (%)	9.1 ± 4.6	15.9 ± 2.9	7.2 ± 3.3

[1] Data is presented as mean ± standard deviation

[2] A total of 11 subjects (n=5 nonsmokers, n=6 smokers) was used for RNA-Seq analysis of gene expression. An independent sample of n=27 was used for microarray analysis

[3] B = black, W = white, O = other.

[4] Pulmonary function testing parameters are given as % of predicted value with the exception of FEV1/FVC, which is reported as % observed; FVC - forced vital capacity, FEV1 - forced expiratory volume in 1 sec, TLC - total lung capacity, DLCO - diffusing capacity of the lungs for carbon monoxide.

[5] From cytospins of small airway epithelium brushings.

[6] As a % of small airway epithelium recovered.

Additional Table S2. Mapping Summary[1]

Subject		Total number of reads	Pass filter / align bowtie (%)[2]	Relative %		
				Exonic	Intronic	Intergenic
Nonsmoker	1	1.74×10^7	86	54	21	24
	2	1.72×10^7	85	56	18	26
	3	1.59×10^7	86	63	13	24
	4	1.98×10^7	82	59	20	22
	5	1.77×10^7	88	58	18	24
Smoker	1	1.58×10^7	84	59	16	25
	2	1.52×10^7	85	53	17	30
	3	1.42×10^7	74	53	21	26
	4	1.66×10^7	82	52	22	26
	5	1.30×10^7	88	54	16	30
	6	1.92×10^7	80	56	20	24
Mean ± SD		$1.65 \times 10^7 \pm 2.01 \times 10^6$	84 ± 4	56 ± 3	18 ± 2	26 ± 3

[1] Summary of mRNA-Seq read counts and mapping statistics for all samples of small airway epithelium (healthy nonsmokers, n=5; healthy smokers, n=6).

[2] Percent of reads with 2 or less mismatches aligned to the reference genome build UCSC hg19 using Bowtie v 0.12 out of the total number of reads generated by Illumina GAII.

Additional Table S3. Comparison of the Median Expression Levels of Different Categories of Genes in the Small Airway Epithelium of Healthy Nonsmokers and Healthy Smokers[1]

	Ubiquitous[2]								Small airway epithelium-enriched[2]							
	Nonsmoker				Smoker				Nonsmoker				Smoker			
		#(%)				#(%)				#(%)				#(%)		
Category	Median	Low	Med	High	Median	Low	Med	High	Median	Low	Med	High	Median	Low	Med	High
All	8.6	147(1.9)	4132(54.3)	3328(43.7)	8.3	165(2.2)	4175(54.9)	3267(42.9)	1.8	2995(36.2)	3859(46.7)	1416(17.1)	1.6	3101(37.5)	3835(46.4)	1334(16.1)
Transcription	8.8	8(1.1)	403(56.1)	308(42.8)	8.4	11(1.5)	400(55.6)	308(42.8)	1.8	126(36.8)	178(52.1)	38(11.1)	1.6	127(37.1)	178(52.0)	37(10.8)
Translation	21.7	1(0.4)	77(28.8)	189(70.8)	19.9	1(0.4)	80(30.0)	186(69.7)	5.6	9(17.3)	22(42.3)	21(40.4)	5.1	9(17.3)	26(50.0)	17(32.7)
Immunity	10.2	4(4.3)	42(45.2)	47(50.5)	10.3	4(4.3)	40(43.0)	49(52.7)	1.4	114(41.9)	123(45.2)	35(12.9)	1.4	111(40.8)	132(48.5)	29(10.7)
Signal transduction	8.3	15(2.3)	363(55.2)	280(42.5)	8.0	15(2.3)	359(54.6)	284(43.2)	1.2	325(45.8)	305(43.0)	79(11.2)	1.1	328(46.3)	302(42.6)	79(11.1)
Adhesion	9.2	2(1.7)	58(48.7)	59(49.6)	10.4	4(3.4)	53(44.5)	62(52.1)	1.2	93(46.5)	87(43.5)	20(10.0)	1.1	95(47.5)	86(43.0)	19(9.5)
Membrane receptors	7.9	4(3.8)	55(52.9)	45(43.3)	8.7	5(4.8)	51(49.0)	48(46.2)	1.1	127(48.3)	104(39.5)	32(12.2)	1.0	127(48.3)	106(40.3)	30(11.4)
Ion transporters	9.2	6(3.6)	80(48.5)	79(47.9)	9.1	5(3.0)	84(50.9)	76(46.1)	1.4	109(43.8)	102(41.0)	38(15.3)	1.2	111(44.6)	99(39.8)	39(15.7)

[1] See Figure 3 for distribution plots within each category.

[2] For ubiquitous and small airway epithelium-enriched genes divided by functional category, the median RPKM, number of genes (#) and % by expression level grouping are presented for nonsmokers and smokers.

Additional Table S4. Cell Type-specific Gene Lists

	Gene symbols											
**Ciliated cells[1]**	ACTG1	AK5	ARL6	BBS1	BBS10	BBS2	BBS4	BBS5	BBS7	BBS9	CALM3	CCDC146
	CCDC28B	CCDC63	CETN3	CROCC	CYS1	DNAH5	DNAH7	DNAH9	DNAI1	DNAI2	DNAL1	DNALI1
	DYNC2H1	DYNLL2	DYNLRB2	DYNLT1	EFHC1	GAS8	GLI2	HSPA1A	HSPA1B	HTR1B	HTR2C	HYDIN
	IFT122	IFT140	IFT172	IFT20	IFT52	IFT57	IFT74	IFT80	IFT81	IFT88	KIF3A	KIF3B
	KIFAP3	LRRCC1	MKKS	MKS1	OFD1	PDGFRA	PKD2	PKHD1	PPP1CC	PPP2R1A	RIBC1	RSPH3
	RSPH4A	SMO	SPAG6	SSNA1	TCTEX1D2	TEKT2	TEKT3	TMEM67	TTC8	TUBA1A	TUBB2C	TUBE1
	TUBG1	WDR63	WDR78									
**Secretory cells[2]**	EMCN	EMR1	EMR2	EMR3	GCNT3	MUC1	MUC12	MUC13	MUC15	MUC16	MUC17	MUC2
	MUC20	MUC4	MUC5B	MUC6	MUC7	MUCL1	PARM1	TFF1	TFF3	TIMD4		
**Neuroendocine cells[3]**	ASCL1	CALCB	CHGA	ENO2	GRP	PENK	SCG2	TAC1	UCHL1			
**Basal cells[4]**	A2ML1	AAK1	ABCA12	ABCC3	ACBD6	ACOT7	ACSL1	ACSL4	ACTN1	ADA	ADAM17	ADAM8
	ADAM9	ADAMTS1	ADCY7	ADK	ADM	ADRB2	AEN	AGAP3	AGFG1	AHNAK2	AIDA	AIF1L
	AIM1L	AK3	AK3L1	AKAP12	AKIRIN2	AKR1B1	AKR1B10	AKT3	ALDH1A3	ALDH1B1	ALDH1L2	ALDOA
	ALOX15B	ALOXE3	ALS2CL	AMIGO2	AMMECR1	AMOTL1	AMOTL2	ANAPC11	ANGPTL4	ANKRD11	ANKRD29	ANKRD57
	ANKRD9	ANXA3	AOX1	AP1S1	AP1S3	AP2B1	AP2S1	AP3S1	APBB2	APOOL	AREG	AREGB
	ARG2	ARHGAP10	ARHGAP29	ARHGEF10	ARID3A	ARID3B	ARL5B	ARL8B	ARMET	ARNTL2	ARPC2	ARPC5L
	ARSJ	ARTN	ASAP1	ASNS	ASPH	ATAD2	ATF4	ATL3	ATP5SL	AVEN	AVPR1B	AXL
	B3GALT6	BACH1	BCAT1	BCL10	BEX2	BICD1	BICD2	BLVRB	BMP1	BMP2	BNC1	BNIP2
	BNIP3	BOK	BOP1	BRI3	BVES	C10orf12	C10orf47	C11orf24	C11orf73	C12orf29	C12orf39	C12orf5
	C12orf54	C14orf34	C15orf23	C16orf5	C16orf74	C17orf39	C17orf45	C17orf91	C18orf19	C18orf54	C19orf10	C19orf48
	C1orf116	C1orf161	C1orf2	C1QBP	C20orf199	C3orf21	C3orf23	C4orf32	C6orf105	C6orf115	C6orf129	C6orf141
	C6orf48	C6orf62	C9orf150	C9orf167	C9orf30	C9orf40	CA12	CA2	CA5BP	CALD1	CALU	CAMK2N1
	CAMKK2	CAMP	CAMSAP1L1	CAP1	CAPG	CAPN14	CAPNS2	CARD10	CAV1	CAV2	CBL	CBLC
	CBS	CBX4	CCBE1	CCDC124	CCDC3	CCDC50	CCDC88A	CCK	CCNA1	CCNB1IP1	CCND2	CCNE1
	CCPG1	CCRN4L	CD109	CD3EAP	CD44	CDC25B	CDC42	CDC42EP1	CDC6	CDCA2	CDCP1	CDH11
	CDH13	CDH3	CDH4	CDK6	CDK8	CDKN1A	CDKN3	CDR2L	CDV3	CEBPG	CENPN	CENPV
	CERCAM	CERK	CHAC1	CHAC2	CHEK1	CHM	CHML	CHST1	CKAP4	CLDND1	CLEC16A	CLGN
	CLIC4	CLIP2	CLIP4	CLTB	CNIH4	CNTN1	COL12A1	COL17A1	COL4A1	COL4A2	COL4A6	COL7A1
	CORO1C	COTL1	CPM	CPS1	CPSF4	CRABP2	CRCT1	CREB5	CRK	CRTAP	CRYAB	CSGALNACT2
	CSNK1E	CSNK2A1	CSPG4	CSRP2	CST3	CSTA	CTH	CTPS	CTSL2	CTTN	CUGBP2	CUL4A
	CYP1B1	CYP51A1	DBF4	DBN1	DCBLD1	DCBLD2	DCUN1D5	DDX10	DDX21	DEFB126	DENND5B	DFNA5
	DHCR7	DIAPH1	DIP2A	DKK1	DKK3	DLGAP4	DNAH14	DNAJB9	DNAJC21	DNM1L	DNM2	DOCK5
	DPH3	DPYSL4	DR1	DRAP1	DSC2	DSC3	DSG2	DSG3	DSP	DST	DTD1	DUSP5
	DUSP6	DUSP7	E2F3	E2F7	EBP	ECM1	EDEM1	EDN1	EGFR	EHD2	EI24	EIF1AX
	EIF2AK4	EIF2S1	EIF2S2	EIF3M	EIF4EBP1	EIF5A	EIF5A2	ELF4	ELL2	ELOVL4	ELOVL5	ELOVL6
	ELOVL7	EMP1	ENO1	EPB41L5	EPHB2	EPHB4	EREG	ERO1L	ERRFI1	ESRP1	ETF1	ETHE1
	ETS1	ETV4	ETV5	FABP5	FADS1	FADS2	FADS3	FAM110C	FAM120C	FAM126A	FAM127A	FAM127B
	FAM128A	FAM128B	FAM132B	FAM33A	FAM38A	FAM40B	FAM46B	FAM83A	FAM83B	FAM83G	FAM87B	FANCE

Gene symbols											
FAR1	FAT1	FBLIM1	FBN2	FCRLB	FER	FERMT1	FERMT2	FEZ1	FGD1	FGD6	FGF11
FGF2	FHL1	FHL2	FHL3	FIBIN	FIGN	FILIP1L	FJX1	FKBP14	FKBP1B	FKBP5	FLII
FLJ25006	FLJ35024	FLNA	FLNB	FLYWCH1	FNDC3B	FOLR3	FOSL1	FOXA2	FOXD1	FOXL2	FOXN2
FOXQ1	FRAS1	FRMD5	FRMD6	FSCN1	FST	FSTL3	FTSJ1	FXYD5	FYN	G0S2	GABPB1
GADD45A	GAL	GALNT10	GALNT14	GALNT5	GARS	GART	GAS5	GATAD2A	GDE1	GDF15	GIGYF2
GJA1	GJB2	GJB3	GJB6	GLB1L3	GLIPR1	GLIS2	GLRX2	GLRX3	GLS	GLT8D2	GM2A
GMFB	GMPS	GNA15	GNAI3	GNB1	GNG10	GOLT1B	GPC1	GPR115	GPR126	GPR153	GPR37
GPR87	GPRC5A	GPSM1	GPX3	GRB10	GRB14	GRN	GRPEL2	GTF2F2	GTPBP2	GTPBP4	H1F0
H2AFZ	HAUS6	HBEGF	HCFC1R1	HDGFRP3	HEATR7A	HES2	HIF1A	HK2	HKDC1	HMGA1	HMGA2
HMGB3	HMGB3L1	HMGN4	HN1	HOMER1	HOMER3	HOPX	HRH1	HSD11B1	HSD17B1	HSD17B2	HSF2BP
HSP90B1	HSPA13	HSPA5	HSPA9	HSPB8	HSPC159	HSPG2	HTR7	IBTK	ID3	IER3	IER5
IFNE	IFRD1	IFRD2	IGF2R	IGFBP6	IGFL1	IL13RA2	IL18	IL1B	IL1RAP	IL1RL1	IL1RN
IL20RB	IL24	INF2	INHBE	INPP4B	INPP5D	IRAK1	IRS1	ITCH	ITGA3	ITGA5	ITGA6
ITGB1	ITGB4	ITGB6	ITPK1	ITPR3	ITPRIP	IVL	JAG1	JARID2	JOSD1	JUB	JUP
KCMF1	KCNG1	KCNK6	KCNQ5	KCTD9	KDELR2	KIAA0323	KIAA0802	KIAA1128	KIAA1804	KIAA1949	KIF13A
KIF1B	KIF21B	KIRREL	KLC3	KLF13	KLF6	KLF7	KLF8	KLF9	KLHL29	KLHL7	KLK10
KLK5	KLK6	KLK7	KLK8	KPNA2	KPNB1	KRT16	KRT17	KRT34	KRT5	KRT6A	KRT6B
KRT7	KRTAP2-4	LAD1	LAMA3	LAMB1	LAMB3	LAMC1	LAMC2	LAMP3	LARP2	LATS2	LDHA
LDLR	LDLRAD3	LEMD1	LEPR	LEPREL1	LEPREL2	LGALS1	LHFP	LHFPL2	LIPG	LMNA	LOC100127983
LOC100128501	LOC100129105	LOC100130938	LOC100131262	LOC136242	LOC147645	LOC221710	LOC284561	LOC284889	LOC338620	LOC339400	LOC344887
LOC401074	LOC401317	LOC440731	LOC440894	LOC441528	LOC554202	LOC646470	LOC653110	LOC728344	LOC729082	LOC729680	LOC96610
LOXL2	LPAR5	LPCAT1	LRP12	LRRC42	LRRC59	LRRC8A	LRRC8C	LRRFIP2	LTBP1	LUZP1	LYPD3
LZIC	MAD2L2	MALAT1	MALL	MALT1	MAN2A1	MAP1LC3B	MAP2	MAP2K1	MAP3K9	MAP4	MAP4K4
MAP7D1	MAPK13	MAPK6	MAPKAP1	MAPKAPK3	MARK1	MARS	MATN3	MBD1	MBOAT2	MCAM	MCART1
MCC	MCFD2	MCOLN3	MCTP1	ME1	MED14	MED8	MET	METTL11A	METTL8	MEX3C	MFAP5
MFHAS1	MFI2	MGAT4B	MGC39372	MGC87042	MGST1	MIB1	MICA	MICAL2	MICALL1	MICALL2	MICB
MKKS	MKNK2	MLLT11	MMD	MMP1	MMP2	MMP28	MN1	MOCOS	MORC4	MPHOSPH6	MPP6
MRC2	MREG	MRPL52	MRPS12	MRPS15	MRPS16	MRPS22	MSN	MT1E	MT1F	MT1G	MT1H
MT1P2	MT1X	MT2A	MTDH	MTHFD1L	MTHFD2	MTP18	MXD1	MYC	MYL12A	MYO10	MYO1B
MYO1C	MYO5A	NAGS	NAMPT	NAP1L1	NAPRT1	NCKAP1	NCOA7	NCRNA00084	NDE1	NDRG1	NDST1
NDUFA4L2	NDUFAF2	NEDD4	NENF	NETO2	NF2	NFIL3	NGFRAP1	NIPAL1	NIPAL4	NLN	NLRP1
NME1	NME4	NMU	NOP16	NP	NPAS2	NPC1	NPM1	NRAS	NRBF2	NRG1	NRP1
NT5DC2	NT5E	NTAN1	NTN4	NTNG1	NUDT11	NUPL1	OBFC2A	OCIAD2	ODC1	OGFRL1	OLA1
OLAH	OPN3	OSMR	OSTC	OSTM1	OTOS	OXSR1	PABPC4	PACSIN3	PAICS	PARD6G	PARVB
PAWR	PCK2	PCNXL2	PCSK9	PCTK1	PDCD5	PDE5A	PDGFA	PDGFC	PDIA6	PDK1	PDLIM5
PDLIM7	PDS5A	PDXK	PDZD8	PEA15	PEAR1	PEG10	PFDN2	PFDN4	PFKFB4	PFN1	PGAM1
PGAM5	PGF	PGK1	PGM2	PGM3	PHACTR3	PHF19	PHGDH	PHLDA1	PHLDA2	PHLDB2	PITPNC1
PKP1	PKP2	PLA2G12A	PLAC1	PLAU	PLAUR	PLCD3	PLCH2	PLEC1	PLEK2	PLEKHA1	PLEKHG3
PLK3	PLOD2	PLS3	PLSCR3	PMAIP1	PMEPA1	POPDC3	PORCN	PPARD	PPARG	PPAT	PPFIBP1
PPP1R13L	PPP1R14A	PPP1R14B	PPP2R5C	PPP4R4	PPPDE1	PPPDE2	PRAGMIN	PREP	PRKCDBP	PRNP	PROCR
PRPH	PRRG1	PRSS1	PRSS2	PRSS3	PRSS8	PSAT1	PSMA7	PSMD12	PSMG1	PSORS1C1	PSTPIP2
PTEN	PTGFRN	PTGS2	PTHLH	PTK6	PTP4A1	PTPLA	PTPLB	PTPN11	PTPN12	PTPN14	PTPRG
PTRF	PTRH2	PTTG3	PVR	PVRL1	PXDN	PYCR1	PYGB	PYY	QKI	QPCT	R3HDM1
RAB27B	RAB32	RAB38	RAB7L1	RABGGTB	RAC2	RALA	RANGRF	RAP1GDS1	RAP2A	RAP2B	RAP2C

Gene symbols											
RBCK1	RBM9	RBMS2	RCOR1	RDH11	REEP4	RGS10	RGS16	RGS20	RHCG	RHEB	RHOBTB2
RHOC	RHOD	RHOF	RIOK3	RIPK2	RNASE7	RNF165	RNMT	ROBO3	RP5-1022P6.2	RP6-213H19.1	RPE
RPL10	RPL10L	RPL15P22	RPL22L1	RPL26P37	RPL37	RPL7AP36	RPS10P5	RPS17P5	RPS2	RPS21	RPS28P6
RRAS	RRAS2	RRP1B	RSL1D1	RSL24D1	RSRC1	RSU1	RTKN	RTTN	S100A10	S100A14	S100A16
S100A2	S100A9	SACS	SAMD4A	SAMD5	SARS	SBSN	SC4MOL	SC65	SCARB1	SCD	SCEL
SCG5	SCHIP1	SCLT1	SCRIB	SDC1	SDCBP2	SDSL	SEC14L2	SEC23A	SEC61G	SEH1L	SEL1L
SEMA3B	SEMA3F	SERINC2	SERPINB2	SERPINB5	SERPINB7	SERPINB8	SERPINE1	SERPINE2	SERPINH1	SESN2	SF3A2
SFN	SFRS12IP1	SFTA1P	SFXN1	SGCG	SGPP1	SGTB	SH2D5	SH3BGRL3	SH3BP2	SH3D20	SH3RF2
SH3TC1	SHC1	SHMT2	SIGMAR1	SLC10A3	SLC16A1	SLC16A2	SLC16A3	SLC16A4	SLC22A3	SLC25A15	SLC25A43
SLC2A1	SLC2A9	SLC35E4	SLC35F2	SLC38A1	SLC38A2	SLC38A5	SLC39A14	SLC3A2	SLC4A5	SLC4A7	SLC6A15
SLC6A8	SLC7A1	SLC7A11	SLC7A5	SLC9A1	SLCO1B3	SMOC1	SMOX	SNAI2	SNCG	SNHG1	SNX24
SNX5	SOCS2	SOX15	SOX4	SOX7	SPAG9	SPCS3	SPIRE1	SPOCD1	SPRR1A	SPRR1B	SPRR2B
SPRR2C	SPRR3	SPRY4	SPTBN1	SQRDL	SQSTM1	SRM	SRP72	SRPX	SRXN1	SSH1	SSR1
SSR3	STARD4	STC2	STK17A	STK32A	STRAP	SUV420H1	SYNGR3	TACC1	TAF1D	TANC2	TARS
TATDN1	TAX1BP3	TBC1D2	TBC1D7	TBRG4	TCEA1	TCF3	TCP11L1	TEAD2	TEAD4	TES	TFAP2A
TFPI2	TGFA	TGFB1	TGFB1I1	TGFB2	TGFBI	TGFBR1	TGM1	THBD	THBS1	TICAM2	TIMP1
TIMP2	TIMP3	TINAGL1	TJP1	TKT	TLE1	TM4SF1	TM6SF1	TMBIM1	TMEFF1	TMEM158	TMEM167A
TMEM206	TMEM22	TMEM38B	TMEM40	TMIE	TMOD3	TNC	TNFRSF10A	TNFRSF10B	TNFRSF10D	TNFRSF12A	TNNT1
TNS4	TOR1AIP2	TP63	TPBG	TPD52L1	TPM1	TPM4	TPMT	TPP1	TRAF3IP2	TRAF3IP3	TRAM2
TRIB3	TRIM5	TRIP10	TSEN15	TSPAN4	TSPAN5	TSR1	TUBA4A	TUBB6	TUBGCP3	TUFT1	TWIST2
TXNDC17	UBE2F	UBE2K	UBE2V2	UBLCP1	UBQLN1	UCHL1	UCHL3	UCK2	ULBP2	UNC5B	UPP1
USP3	UST	UTP15	VCL	VDR	VEGFA	VEGFC	VGLL1	VPS37A	VPS4A	WASF1	WBP4
WBP5	WDR41	WDR67	WHSC1	WIZ	WNT7A	WSB2	WWC2	XDH	XPOT	YARS	YDJC
YES1	YIF1B	YKT6	YRDC	ZAK	ZC3H15	ZDHHC12	ZDHHC14	ZDHHC3	ZDHHC9	ZFAND2A	ZMYND11
ZNF185	ZNF215	ZNF281	ZNF598	ZNF83	ZPLD1	ZXDB	ZYX				

[1] List includes genes known to be enriched in expression in ciliated cells [1].

[2] Secretory cell gene lists consisting of all mucins, mucin components and transcription factors.

[3] Neuroendocrine cells [2].

[4] Basal cells [3].

References

1. Dvorak A, Tilley AE, Shaykhiev R, Wang R, Crystal RG: **Do Airway Epithelium Air-liquid Cultures Represent the In Vivo Airway Epithelium Transcriptome?** *Am J Respir Cell Mol Biol* 2010, **44:** 465-473.
2. Carolan BJ, Harvey BG, De BP, Vanni H, Crystal RG: **Decreased expression of intelectin 1 in the human airway epithelium of smokers compared to nonsmokers.** *J Immunol* 2008, **181:** 5760-5767.
3. Hackett NR, Shaykhiev R, Walters MS, Wang R, Zwick RK, Ferris B *et al.*: **The human airway epithelial basal cell transcriptome.** *PLoS One* 2011.

Additional Table S5. Reproducibility of Smoking-responsive Genes Discovered by Microarray Using RNA-Seq Method[1]

ProbeSetID	Gene title	Micorarray p value	Micorarray Ratio (S/NS)	RNA-Seq p value	RNA-Seq Ratio (S/NS)
206561_s_at	AKR1B10	<0.0001	56.62	0.0222	58.45
202436_s_at	CYP1B1	<0.0001	55.00	0.4215	75.52
202437_s_at	CYP1B1	<0.0001	47.95	0.4215	75.52
205749_at	CYP1A1	0.0114	35.18	0.5101	405.52
202435_s_at	CYP1B1	<0.0001	34.67	0.4215	75.52
201387_s_at	UCHL1	<0.0001	21.19	0.3822	5.87
217678_at	SLC7A11	<0.0001	14.67	0.1651	14.12
205278_at	GAD1	0.0025	14.53	0.7536	2.45
209921_at	SLC7A11	<0.0001	10.68	0.1651	14.12
205623_at	ALDH3A1	<0.0001	9.82	0.1610	8.50
223122_s_at	SFRP2	0.0071	9.66	0.2589	9.45
224279_s_at	CABYR	<0.0001	9.36	0.1710	10.10
210505_at	ADH7	0.0001	8.75	0.1574	6.37
209875_s_at	SPP1	0.0021	8.54	0.0474	8.66
202831_at	GPX2	<0.0001	7.90	0.0002	6.73
217626_at	AKR1C1	0.0002	7.20	0.2492	5.50
233076_at	JAKMIP3	0.0137	6.92	0.0002	4.32
223121_s_at	SFRP2	0.0335	6.90	0.2589	9.45
1555854_at	AKR1C2	<0.0001	6.89	0.0033	7.93
219928_s_at	CABYR	<0.0001	6.73	0.1710	10.10
1553602_at	MUCL1	0.0002	6.44	0.2717	5.26
204058_at	ME1	<0.0001	5.37	0.2217	4.75
208596_s_at	UGT1A10	0.0024	5.12	0.2112	2.57
204059_s_at	ME1	<0.0001	5.10	0.2217	4.75
209699_x_at	AKR1C2	<0.0001	5.03	0.0033	7.93
215125_s_at	UGT1A5	0.0017	4.95	0.1215	6.30
201468_s_at	NQO1	<0.0001	4.76	0.2952	4.55
206254_at	EGF	0.0017	4.56	0.0389	5.13
210769_at	CNGB1	0.0056	4.55	0.0206	10.23
239595_at	GPX2	0.0003	4.40	0.0002	6.73
1559072_a_at	ELFN2	0.0065	4.38	0.1507	12.42
206515_at	CYP4F3	0.0004	4.38	0.3743	3.75
207126_x_at	UGT1A8	0.0004	4.35	0.7432	1.61
201467_s_at	NQO1	<0.0001	4.20	0.2952	4.55
206094_x_at	UGT1A3	0.0013	4.13	0.0687	2.55
216594_x_at	AKR1C1	<0.0001	3.99	0.2492	5.50
204532_x_at	UGT1A4	0.0011	3.97	0.4936	1.36
209160_at	AKR1C3	0.0001	3.96	0.0132	4.28
206153_at	CYP4F11	0.0463	3.86	0.0516	2.92

		Micorarray		RNA-Seq	
ProbeSetID	Gene title	p value	Ratio (S/NS)	p value	Ratio (S/NS)
239229_at	PHEX	0.0114	3.79	0.0098	6.29
211653_x_at	AKR1C2	<0.0001	3.78	0.0033	7.93
229354_at	AHRR	0.0054	3.71	0.0062	19.70
224997_x_at	H19	0.0404	3.53	0.4395	8.45
210519_s_at	NQO1	<0.0001	3.52	0.2952	4.55
207469_s_at	PIR	<0.0001	3.47	0.0981	3.41
205221_at	HGD	0.0165	3.44	0.4270	3.68
204151_x_at	AKR1C1	<0.0001	3.31	0.2492	5.50
236884_at	RIMKLA	0.0311	3.28	0.9483	2.00
205413_at	MPPED2	0.0139	3.17	0.7816	1.65
217997_at	PHLDA1	0.0015	2.98	0.0367	3.00
1562102_at	AKR1C1	0.0389	2.96	0.2492	5.50
210397_at	DEFB1	0.0173	2.88	0.5010	3.66
225842_at	PHLDA1	0.0216	2.85	0.0367	3.00
204341_at	TRIM16	0.0008	2.78	0.9763	2.03
219475_at	OSGIN1	0.0483	2.76	0.2170	5.85
233202_at	CNTNAP3	0.0317	2.75	0.3610	1.32
226425_at	CLIP4	0.0106	2.74	0.6687	2.36
1558738_at	NOL3	0.0386	2.64	0.9049	1.66
205379_at	CBR3	0.0127	2.63	0.0616	2.90
205328_at	CLDN10	0.0299	2.60	0.0140	2.55
230030_at	HS6ST2	0.0270	2.57	0.0182	1.58
220197_at	ATP6V0A4	0.0279	2.49	0.4084	2.22
224480_s_at	AGPAT9	0.0407	2.47	0.0003	1.98
217996_at	PHLDA1	0.0270	2.47	0.0367	3.00
208161_s_at	ABCC3	0.0335	2.44	0.4675	3.06
201266_at	TXNRD1	<0.0001	2.41	0.6507	2.44
208700_s_at	TKT	0.0067	2.33	0.6808	2.35
1555330_at	GCLC	0.0250	2.33	0.8495	1.83
1553183_at	UMODL1	0.0466	2.32	0.3210	1.25
209655_s_at	TMEM47	0.0443	2.32	0.0564	1.40
201272_at	AKR1B1	0.0210	2.32	0.0033	2.54
203192_at	ABCB6	0.0002	2.29	0.9834	1.98
225252_at	SRXN1	0.0235	2.29	0.0003	2.97
228205_at	TKT	0.0311	2.28	0.6808	2.35
225609_at	GSR	0.0125	2.14	0.9644	1.92
209448_at	HTATIP2	0.0095	2.14	0.8056	1.78
219958_at	C20orf46	0.0456	2.14	0.0066	3.21
219944_at	CLIP4	0.0093	2.11	0.6687	2.36
226084_at	MAP1B	0.0433	2.11	0.0082	2.03
221908_at	RNFT2	0.0109	2.06	0.0102	2.00

		Micorarray		RNA-Seq	
**ProbeSetID**	**Gene title**	**p value**	**Ratio (S/NS)**	**p value**	**Ratio (S/NS)**
224772_at	NAV1	0.0483	2.05	0.0261	1.87
219884_at	LHX6	0.0061	2.04	0.7702	2.49
210253_at	HTATIP2	<0.0001	2.04	0.8056	1.78
201463_s_at	TALDO1	0.0007	2.00	0.9590	2.06
223796_at	CNTNAP3	0.0309	1.98	0.3610	1.32
236465_at	RNF175	0.0469	1.89	0.0307	2.29
200924_s_at	SLC3A2	0.0469	1.88	0.7816	1.74
1569144_a_at	C9orf169	0.0415	1.88	0.1960	1.03
212850_s_at	LRP4	0.0483	1.87	0.0183	1.60
230323_s_at	TMEM45B	0.0415	1.86	0.6897	1.51
222016_s_at	ZNF323	0.0219	1.82	0.8543	1.51
203925_at	GCLM	0.0095	1.77	0.0125	1.90
229523_at	TMEM200C	0.0012	1.75	0.8426	1.54
1558290_a_at	PVT1	0.0138	1.74	0.0418	1.55
202923_s_at	GCLC	0.0038	1.73	0.8495	1.83
242478_at	GSTA1	0.0170	1.71	0.5136	1.47
202922_at	GCLC	0.0114	1.70	0.8495	1.83
221823_at	C5orf30	0.0433	1.69	0.9464	1.46
59625_at	NOL3	0.0159	1.69	0.9049	1.66
228754_at	SLC6A6	0.0363	1.68	0.4237	1.42
212221_x_at	IDS	0.0156	1.67	0.4079	1.41
206822_s_at	L3MBTL	0.0415	1.66	0.1613	1.07
212233_at	MAP1B	0.0116	1.64	0.0082	2.03
202804_at	ABCC1	0.0268	1.61	0.4896	1.42
229860_x_at	C4orf48	0.0327	1.61	0.0187	1.41
204970_s_at	MAFG	0.0350	1.58	0.5221	1.84
218684_at	LRRC8D	0.0415	1.50	0.4754	1.45
226798_at	BCL2L13	0.0279	1.44	0.1160	1.26
217994_x_at	CPSF3L	0.0294	0.77	0.0081	0.82
35150_at	CD40	0.0415	0.75	0.1599	0.82
204102_s_at	EEF2	0.0311	0.75	0.0055	0.75
204218_at	C11orf51	0.0309	0.75	0.1363	0.94
212540_at	CDC34	0.0439	0.74	0.0359	0.94
227374_at	EARS2	0.0433	0.72	0.0921	0.83
227108_at	STARD9	0.0142	0.72	0.0097	0.82
231713_s_at	ELP2	0.0404	0.72	0.0159	0.85
228346_at	ZNF844	0.0389	0.71	0.0256	0.89
224210_s_at	PXMP4	0.0415	0.71	0.3248	0.85
221792_at	RAB6B	0.0438	0.70	0.5221	0.51
226874_at	KLHL8	0.0380	0.69	0.8859	0.82
205896_at	SLC22A4	0.0245	0.69	0.0013	0.67

ProbeSetID	Gene title	Micorarray		RNA-Seq	
		p value	Ratio (S/NS)	p value	Ratio (S/NS)
229693_at	TMEM220	0.0415	0.68	0.0173	0.82
227041_at	SESTD1	0.0117	0.68	0.0141	0.81
212061_at	SR140	0.0415	0.68	0.0654	0.98
213900_at	FAM189A2	0.0180	0.68	<0.0001	0.52
202854_at	HPRT1	0.0221	0.68	0.0042	0.74
204608_at	ASL	0.0439	0.67	0.0021	0.70
204546_at	KIAA0513	0.0210	0.67	0.0054	0.85
229356_x_at	INO80	0.0439	0.67	0.0043	0.74
227388_at	TUSC1	0.0438	0.66	0.0001	0.70
209356_x_at	EFEMP2	0.0117	0.66	0.0740	0.89
201194_at	SEPW1	0.0231	0.66	0.0154	0.81
234339_s_at	GLTSCR2	0.0470	0.65	0.0099	0.77
230054_at	PRRT1	0.0244	0.65	0.0777	0.81
227197_at	SGEF	0.0108	0.65	0.0058	0.73
217478_s_at	HLA-DMA	0.0056	0.65	0.1095	0.73
228051_at	KIAA1244	0.0309	0.65	0.0067	0.77
221534_at	C11orf68	0.0119	0.65	0.6403	0.73
210473_s_at	GPR125	0.0480	0.65	0.0005	0.63
210347_s_at	BCL11A	0.0443	0.64	0.8850	0.88
226216_at	INSR	0.0244	0.64	0.0083	0.75
202510_s_at	TNFAIP2	0.0447	0.64	0.0029	0.69
224882_at	ACSS1	0.0433	0.64	0.0030	0.73
209043_at	PAPSS1	0.0071	0.64	0.0005	0.63
210166_at	TLR5	0.0022	0.64	<0.0001	0.57
238575_at	OSBPL6	0.0415	0.64	0.0020	0.65
218822_s_at	NPEPL1	0.0238	0.64	0.1936	0.82
226438_at	SNTB1	0.0054	0.63	0.0065	0.75
226747_at	TXNDC16	0.0427	0.63	0.0717	0.71
226197_at	AR	0.0368	0.63	0.1276	0.68
205632_s_at	PIP5K1B	0.0125	0.63	0.0002	0.52
209737_at	MAGI2	0.0368	0.63	0.1147	0.86
201136_at	PLP2	0.0350	0.63	0.0055	0.75
226213_at	ERBB3	0.0449	0.63	0.0014	0.74
224901_at	SCD5	0.0206	0.63	0.0006	0.58
201397_at	PHGDH	0.0479	0.62	0.0681	0.62
204099_at	SMARCD3	0.0303	0.62	0.2503	0.72
204065_at	CHST10	0.0166	0.62	0.0002	0.56
1558304_s_at	TSEN54	0.0287	0.62	0.0079	0.81
226364_at	HIP1	0.0433	0.61	0.0993	0.74
212646_at	RFTN1	0.0138	0.61	<0.0001	0.60
210963_s_at	GYG2	0.0439	0.61	0.7055	0.62

		Micorarray		RNA-Seq	
ProbeSetID	Gene title	p value	Ratio (S/NS)	p value	Ratio (S/NS)
222912_at	ARRB1	0.0245	0.61	0.0013	0.68
209615_s_at	PAK1	0.0324	0.61	0.0026	0.75
220766_at	BTG4	0.0281	0.61	0.0170	0.63
219123_at	ZNF232	0.0279	0.60	0.0076	0.80
228573_at	ANTXR2	0.0003	0.60	0.6845	0.65
225524_at	ANTXR2	0.0024	0.60	0.6845	0.65
205771_s_at	AKAP7	0.0478	0.60	0.1241	0.84
203276_at	LMNB1	0.0045	0.59	0.0791	0.58
207836_s_at	RBPMS	0.0368	0.59	0.0027	0.71
1555793_a_at	ZFP82	0.0166	0.59	0.1539	0.59
201866_s_at	NR3C1	0.0388	0.59	0.0670	0.82
205880_at	PRKD1	0.0171	0.59	0.0611	0.72
213348_at	CDKN1C	0.0013	0.59	0.3317	0.51
212282_at	TMEM97	0.0350	0.59	0.0005	0.61
219389_at	SUSD4	0.0404	0.58	<0.0001	0.53
229499_at	CAPN13	0.0379	0.58	0.0001	0.57
227405_s_at	FZD8	0.0415	0.58	0.0001	0.46
206004_at	TGM3	0.0415	0.57	0.0884	0.59
227376_at	GLI3	0.0147	0.57	0.0942	0.53
204880_at	MGMT	0.0169	0.57	0.7438	0.82
224046_s_at	PDE7A	0.0439	0.57	0.0033	0.68
214462_at	SOCS6	0.0156	0.56	0.0008	0.71
1556012_at	KLHDC7A	0.0466	0.56	0.0026	0.71
213629_x_at	MT1F	0.0271	0.56	0.8296	0.47
204745_x_at	MT1G	0.0086	0.56	0.0100	0.25
221016_s_at	TCF7L1	0.0279	0.56	0.0055	0.76
223822_at	SUSD4	0.0162	0.55	<0.0001	0.53
241342_at	TMEM65	0.0344	0.55	0.0116	0.70
223593_at	AADAT	0.0015	0.54	0.0002	0.52
202729_s_at	LTBP1	0.0439	0.54	0.2538	0.34
237020_at	TMEM146	0.0331	0.54	0.0006	0.64
1557136_at	ATP13A4	0.0415	0.54	0.7581	0.57
1555095_at	C6orf123	0.0169	0.53	0.0002	0.50
225163_at	FRMD4A	0.0404	0.53	0.0023	0.63
217165_x_at	MT1F	0.0333	0.53	0.8296	0.47
220389_at	CCDC81	0.0299	0.53	0.9124	0.64
215990_s_at	BCL6	0.0433	0.52	0.0007	0.70
221601_s_at	FAIM3	0.0415	0.52	0.0106	0.79
226955_at	AFAP1L1	0.0261	0.52	0.0004	0.57
214290_s_at	HIST2H2AA4	0.0162	0.52	0.0001	0.56
226649_at	PANK1	0.0391	0.52	0.2618	0.39

		Micorarray		RNA-Seq	
**ProbeSetID**	**Gene title**	**p value**	**Ratio (S/NS)**	**p value**	**Ratio (S/NS)**
223495_at	CCDC8	0.0117	0.51	0.0006	0.59
227702_at	CYP4X1	0.0483	0.51	0.0002	0.45
232481_s_at	SLITRK6	0.0028	0.51	0.0002	0.41
205608_s_at	ANGPT1	0.0095	0.51	0.1548	0.18
205356_at	USP13	<0.0001	0.51	0.3731	0.52
203865_s_at	ADARB1	0.0238	0.50	0.0771	0.57
230121_at	C1orf133	0.0245	0.49	0.0002	0.51
224325_at	FZD8	0.0337	0.49	0.0001	0.46
219489_s_at	NXN	0.0238	0.49	0.0002	0.54
212859_x_at	MT1E	0.0183	0.49	0.0049	0.57
218741_at	CENPM	0.0028	0.49	0.0003	0.53
202357_s_at	CFB	0.0294	0.48	0.0021	0.61
205833_s_at	PART1	0.0443	0.48	0.0079	0.45
224823_at	MYLK	0.0447	0.48	0.8118	0.71
202771_at	FAM38A	0.0093	0.48	0.0014	0.53
236175_at	TRIM55	0.0230	0.47	0.0005	0.51
202936_s_at	SOX9	0.0364	0.46	0.0057	0.61
203680_at	PRKAR2B	0.0206	0.46	0.1869	0.47
228547_at	NRXN1	0.0309	0.46	0.1409	0.42
204179_at	MB	0.0221	0.46	0.1872	0.40
823_at	CX3CL1	0.0388	0.46	0.0002	0.35
228124_at	ABHD12	0.0311	0.45	0.1480	0.87
220013_at	EPHX3	0.0380	0.45	0.3031	0.44
201719_s_at	EPB41L2	0.0447	0.44	0.1486	0.53
225202_at	RHOBTB3	0.0015	0.43	0.0654	0.53
219630_at	PDZK1IP1	0.0466	0.43	0.0018	0.52
223821_s_at	SUSD4	0.0014	0.42	<0.0001	0.53
205279_s_at	GLRB	0.0111	0.41	0.0029	0.43
203060_s_at	PAPSS2	0.0219	0.41	0.0698	0.69
209897_s_at	SLIT2	0.0156	0.41	0.6393	0.44
229281_at	NPAS3	0.0380	0.40	0.5849	0.37
232176_at	SLITRK6	0.0084	0.40	0.0002	0.41
202747_s_at	ITM2A	0.0071	0.39	<0.0001	0.25
212344_at	SULF1	0.0219	0.39	<0.0001	0.37
1553589_a_at	PDZK1IP1	0.0466	0.38	0.0018	0.52
201939_at	PLK2	0.0104	0.38	0.8345	0.43
203939_at	NT5E	0.0075	0.38	<0.0001	0.46
207414_s_at	PCSK6	0.0049	0.38	0.8257	0.35
203027_s_at	MVD	0.0350	0.37	0.0237	0.88
208451_s_at	C4A	0.0284	0.36	0.0008	0.40
230944_at	C6orf223	0.0404	0.36	0.0004	0.22

		Micorarray		RNA-Seq	
ProbeSetID	Gene title	p value	Ratio (S/NS)	p value	Ratio (S/NS)
204041_at	MAOB	0.0012	0.36	<0.0001	0.49
235377_at	C6orf142	0.0404	0.35	0.0867	0.44
41577_at	PPP1R16B	0.0035	0.31	0.0699	0.36
207096_at	SAA4	0.0147	0.30	0.0006	0.24
219410_at	TMEM45A	0.0071	0.29	0.0256	0.41
213258_at	TFPI	0.0075	0.27	0.0751	0.67
217767_at	C3	0.0027	0.25	0.0007	0.35
227450_at	ERP27	0.0016	0.23	0.0773	0.25
219049_at	CSGALNACT1	0.0244	0.23	0.5760	0.48
236420_s_at	ANO4	0.0478	0.22	0.0347	0.30
240699_at	SEC14L3	0.0056	0.21	0.0052	0.43
216346_at	SEC14L3	0.0079	0.18	0.0052	0.43
212750_at	PPP1R16B	0.0172	0.17	0.0699	0.36
202018_s_at	LTF	0.0052	0.16	0.0009	0.20
214456_x_at	SAA1	<0.0001	0.11	0.0005	0.10
223597_at	ITLN1	0.0335	0.10	0.0216	0.05
208607_s_at	SAA2	0.0002	0.10	0.0008	0.10

[1] The table lists all significant genes (Benjamini Hochberg corrected p value <0.05) with > 1.5 fold difference between n=12 smokers and n=12 nonsmokers as determined by mciroarray. For each probeset the corresponding genes was assessed by RNA-Seq for n=5 nonsmokers and n=6 smokers and the p value and fold change returned.

Additional Table S6. Reproducibility of Smoking-responsive Genes Discovered by RNA-Seq Using Microarray Method[1]

Gene title	RNA-Seq p value	RNA-Seq Ratio (S/NS)	Micorarray p value	Micorarray Ratio (S/NS)
AKR1C2	0.0033	7.93	<0.0001	6.89
GPX2	0.0002	6.73	<0.0001	7.90
JAKMIP3	0.0002	4.32	0.0137	6.92
SHCBP1	0.0028	3.60	0.9705	1.06
TRIM16L	0.0007	3.17	0.9887	1.02
SRXN1	0.0003	2.97	0.0235	2.29
STAP1	0.0006	2.82	0.6215	1.39
AKR1B1	0.0033	2.54	0.0210	2.32
G6PD	0.0050	2.50	0.1460	2.34
STRA6	0.0012	2.04	0.4808	1.24
AGPAT9	0.0003	1.98	0.0407	2.47
C1orf51	0.0042	1.98	0.7947	1.12
DIO1	0.0010	1.93	0.0543	2.00
C2orf70	<0.0001	1.86	0.2325	1.60
NPDC1	0.0049	1.85	0.2979	1.59
CHDH	0.0029	1.69	0.8369	1.10
CHST15	0.0015	1.59	0.0933	1.49
IL27RA	0.0017	0.67	0.3188	0.55
CXorf59	0.0012	0.67	0.9031	0.91
CNN3	0.0002	0.66	0.5328	0.73
LPIN1	0.0013	0.66	0.1700	0.79
SEPP1	<0.0001	0.66	0.1699	0.56
ZNF550	0.0002	0.66	0.8482	0.94
ZNF383	0.0007	0.66	0.5822	0.80
NHEDC2	0.0012	0.66	0.9509	0.95
SCNN1G	0.0007	0.66	0.2862	0.58
FGFR2	0.0002	0.66	0.0725	0.50
KIAA1328	0.0002	0.66	0.6438	0.77
WNT5B	0.0007	0.66	0.2423	0.66
HIST2H4A	0.0038	0.66	0.6108	0.81
SEMA4G	0.0009	0.66	0.8996	1.14
SPRY1	0.0049	0.66	0.9419	0.96
TMEM150C	0.0011	0.66	0.5277	0.80
FABP6	0.0017	0.65	0.3201	0.59
PLIN5	0.0019	0.65	0.5028	0.70
GAS6	0.0003	0.65	0.3807	0.71
LMO2	0.0012	0.65	0.6700	0.88
CIITA	0.0020	0.65	0.8731	0.94
CA8	0.0024	0.65	0.1341	0.73
GPX7	0.0029	0.65	0.7227	0.87
CYP2F1	0.0044	0.65	0.5632	0.74
USP51	0.0011	0.65	0.0761	0.65

	RNA-Seq		Micorarray	
**Gene title**	**p value**	**Ratio (S/NS)**	**p value**	**Ratio (S/NS)**
TTC16	<0.0001	0.65	0.3685	0.78
C2orf79	0.0036	0.65	0.2329	0.68
TTYH2	0.0045	0.65	0.9940	0.99
OSBPL6	0.0020	0.65	0.0415	0.64
ACAT2	<0.0001	0.64	0.3298	0.75
IL4R	0.0006	0.64	0.2537	0.68
HNMT	0.0002	0.64	0.1663	0.55
VEPH1	0.0037	0.64	0.5557	0.76
RPLP0	0.0044	0.64	0.3916	0.81
DSG2	0.0029	0.64	0.0775	0.52
PROS1	0.0020	0.64	0.1670	0.63
PPAP2A	0.0048	0.64	0.4481	0.79
KLF13	0.0013	0.64	0.1615	0.71
CDCA4	0.0009	0.64	0.6987	0.90
MFRP	0.0001	0.64	0.2304	0.60
OBSCN	0.0028	0.64	0.9693	1.02
PCDH7	0.0020	0.64	0.3304	0.36
SLC29A3	<0.0001	0.64	0.7531	0.85
TMEM146	0.0006	0.64	0.0331	0.54
PLAGL1	0.0003	0.64	0.2482	0.69
ARNTL	0.0013	0.64	0.2898	0.67
CLDN1	0.0007	0.64	0.5505	0.79
TRMT61A	<0.0001	0.64	0.9042	0.93
FRMD4A	0.0023	0.63	0.0404	0.53
OR7E37P	0.0011	0.63	0.0771	0.70
AMOTL2	0.0047	0.63	0.7126	0.88
POLA2	0.0006	0.63	0.3298	0.73
FLJ39653	0.0050	0.63	0.6763	1.32
PAPSS1	0.0005	0.63	0.0071	0.64
C14orf50	0.0003	0.63	0.0906	0.64
EFEMP1	0.0007	0.63	0.3320	0.62
NTHL1	0.0006	0.63	0.4267	0.76
GPR125	0.0005	0.63	0.0480	0.65
OBSL1	0.0004	0.63	0.1584	0.57
OTUD1	0.0019	0.62	0.5093	0.79
C18orf45	0.0005	0.62	0.3503	0.83
HCP5	0.0032	0.62	0.1695	0.67
SCGB3A1	0.0050	0.62	0.1509	0.75
WDR38	0.0011	0.62	0.0907	0.63
SCARA3	0.0022	0.62	0.1465	0.65
CKB	0.0009	0.62	0.3485	0.69
DEGS2	0.0024	0.61	0.4376	0.67
LYPD6B	0.0006	0.61	0.3653	0.72
TMEM97	0.0005	0.61	0.0350	0.59

	RNA-Seq		Micorarray	
**Gene title**	**p value**	**Ratio (S/NS)**	**p value**	**Ratio (S/NS)**
CFB	0.0021	0.61	0.0294	0.48
LOC100131434	0.0048	0.61	0.4162	0.65
SERPING1	0.0019	0.61	0.1615	0.46
LOC645638	0.0033	0.61	0.1153	0.53
ZNF542	0.0004	0.61	0.3660	0.78
SLAIN1	0.0005	0.61	0.7051	0.81
C11orf95	0.0015	0.61	0.4557	0.76
SLC23A1	0.0012	0.61	0.5616	0.74
CD81	0.0010	0.61	0.3499	0.76
CDT1	0.0003	0.61	0.7311	0.76
KCNB1	0.0035	0.61	0.1755	0.59
CXCL6	0.0008	0.60	0.2493	0.53
LRFN4	<0.0001	0.60	0.8750	0.89
ISLR	0.0033	0.60	0.9494	0.96
RFTN1	<0.0001	0.60	0.0138	0.61
FEZ1	0.0041	0.60	0.2009	0.65
NOTCH3	0.0016	0.59	0.1356	0.59
EFS	0.0010	0.59	0.3642	0.71
SH3RF3	0.0002	0.59	0.1074	0.58
CCDC8	0.0006	0.59	0.0117	0.51
OR2A20P	0.0031	0.59	0.6813	0.82
PDK4	0.0004	0.59	0.0644	0.66
TMEM121	0.0003	0.58	0.1034	0.58
PAPLN	<0.0001	0.58	0.5823	0.83
CDON	0.0006	0.58	0.1733	0.72
SCD5	0.0006	0.58	0.0206	0.63
PLAG1	0.0015	0.58	0.1323	0.58
TAS2R5	0.0047	0.58	0.5911	1.73
CDCA3	0.0040	0.58	0.5381	0.79
ASTN2	0.0005	0.58	0.0997	0.64
LAMB3	0.0009	0.58	0.1153	0.63
KHDRBS3	0.0029	0.58	0.4808	0.80
WFDC6	0.0007	0.58	0.1179	0.57
IFITM3	0.0002	0.58	0.0790	0.50
CLDN16	0.0002	0.58	0.8861	0.84
SEMA3E	0.0014	0.58	0.3865	0.60
PIWIL4	0.0003	0.58	0.5322	0.84
D2HGDH	0.0008	0.58	0.5187	0.72
ZIK1	0.0004	0.58	0.2157	0.66
PLIN2	0.0024	0.57	0.6901	0.76
TLR5	<0.0001	0.57	0.0022	0.64
MT1E	0.0049	0.57	0.0183	0.49
GATSL3	0.0002	0.57	0.6894	0.79
RAMP1	0.0012	0.57	0.6813	0.85

	RNA-Seq		Micorarray	
**Gene title**	**p value**	**Ratio (S/NS)**	**p value**	**Ratio (S/NS)**
CAPN13	0.0001	0.57	0.0379	0.58
AFAP1L1	0.0004	0.57	0.0261	0.52
BCL2	0.0015	0.56	0.3484	0.54
GABRE	0.0008	0.56	0.9165	0.92
HIST2H2AA4	0.0001	0.56	0.0162	0.52
HIST2H2AA3	0.0004	0.56	0.7917	0.89
SULT2B1	0.0022	0.56	0.2039	0.64
CHST10	0.0002	0.56	0.0166	0.62
LPAR5	0.0010	0.56	0.8157	0.86
ADCY2	0.0020	0.56	0.3656	0.66
NEURL1B	0.0002	0.56	0.1392	0.50
EPAS1	0.0014	0.55	0.3264	0.69
EPHA4	0.0005	0.55	0.5983	0.75
FGFR3	0.0042	0.55	0.6813	0.75
ABCA13	0.0002	0.54	0.2581	0.62
LEPR	0.0002	0.54	0.2473	0.39
SLAMF7	0.0021	0.54	0.2576	0.59
SALL2	0.0021	0.54	0.8982	0.93
NXN	0.0002	0.54	0.0238	0.49
IFITM1	0.0002	0.54	0.0999	0.45
OCA2	0.0048	0.53	0.7606	0.84
OLFML1	0.0017	0.53	0.9560	1.08
ITGA9	0.0006	0.53	0.1801	0.47
CENPM	0.0003	0.53	0.0028	0.49
USP27X	0.0001	0.53	0.1890	0.49
SUSD4	<0.0001	0.53	0.0014	0.42
PPAP2B	<0.0001	0.53	0.3104	0.69
FAM38A	0.0014	0.53	0.0093	0.48
AADAT	0.0002	0.52	0.0015	0.54
FAM189A2	<0.0001	0.52	0.0180	0.68
PTGER4	0.0005	0.52	0.3816	0.68
PDZK1IP1	0.0018	0.52	0.0466	0.38
C6orf124	0.0005	0.52	0.4471	0.76
GPR146	0.0010	0.52	0.6860	0.73
DOK7	0.0004	0.52	0.8731	0.93
PIP5K1B	0.0002	0.52	0.0125	0.63
ZNF185	<0.0001	0.52	0.1979	0.72
FADS3	0.0003	0.51	0.2132	0.51
C1orf133	0.0002	0.51	0.0245	0.49
PF4V1	0.0021	0.51	0.3656	0.62
NUAK1	0.0040	0.51	0.9929	0.99
CFD	0.0034	0.51	0.2865	0.53
PCDH20	0.0005	0.51	0.0567	0.47
TRIM55	0.0005	0.51	0.0230	0.47

Gene title	RNA-Seq		Micorarray	
	p value	Ratio (S/NS)	p value	Ratio (S/NS)
FAM20A	<0.0001	0.50	0.3242	0.42
C6orf123	0.0002	0.50	0.0169	0.53
GP5	0.0007	0.50	0.4442	0.75
LOC285735	0.0004	0.49	0.2167	0.63
FHOD3	0.0034	0.49	0.3750	0.61
MAOB	<0.0001	0.49	0.0012	0.36
OPRL1	0.0026	0.48	0.8848	1.16
SERPINB3	0.0043	0.48	0.4081	0.66
SPINLW1	0.0001	0.48	0.0977	0.54
FXYD1	0.0007	0.47	0.3120	0.64
ENPP2	0.0013	0.46	0.2147	0.63
FZD8	0.0001	0.46	0.0337	0.49
CDH11	0.0013	0.46	0.5271	0.64
NEURL3	0.0014	0.46	0.5769	0.79
NT5E	<0.0001	0.46	0.0075	0.38
FLRT3	0.0003	0.46	0.1458	0.57
CALB2	0.0038	0.46	0.5776	0.77
COL9A2	0.0002	0.45	0.2243	0.63
ADAMTS3	0.0007	0.45	0.1420	0.57
CYP4X1	0.0002	0.45	0.0483	0.51
RTN4RL1	0.0003	0.44	0.3400	0.51
EGFL6	0.0047	0.43	0.1460	0.55
GLRB	0.0029	0.43	0.0111	0.41
SLC29A1	<0.0001	0.43	0.1334	0.57
COL6A1	0.0002	0.43	0.6216	0.77
KRT4	0.0026	0.42	0.4859	0.67
SCGB1A1	<0.0001	0.41	0.4670	0.88
C2orf72	0.0017	0.41	0.2267	0.61
SLITRK6	0.0002	0.41	0.0028	0.51
GPR115	0.0004	0.41	0.3148	0.50
C4A	0.0008	0.40	0.0284	0.36
C4B	0.0029	0.40	0.1034	0.56
FAM107A	0.0001	0.39	0.3033	0.77
KCNC4	0.0002	0.38	0.4714	0.75
SULF1	<0.0001	0.37	0.0219	0.39
CX3CL1	0.0002	0.35	0.0388	0.46
C3	0.0007	0.35	0.0027	0.25
WNK4	0.0035	0.34	0.1033	0.26
SNORD89	0.0037	0.34	0.8381	1.12
INMT	0.0002	0.33	0.3947	0.74
C8orf12	0.0018	0.33	0.2519	0.43
SLC14A1	0.0043	0.32	0.9241	0.87
SLCO2A1	<0.0001	0.31	0.2473	0.75
FCGBP	0.0038	0.31	0.0573	0.31

	RNA-Seq		Micorarray	
**Gene title**	**p value**	**Ratio (S/NS)**	**p value**	**Ratio (S/NS)**
GYPC	0.0049	0.30	0.1334	0.18
HSD17B2	0.0002	0.29	0.1155	0.26
SHISA9	<0.0001	0.29	0.2523	0.34
CHI3L1	<0.0001	0.28	0.3104	0.34
ITM2A	<0.0001	0.25	0.0071	0.39
PCDH17	0.0012	0.25	0.0589	0.32
SLC26A4	0.0009	0.24	0.1642	0.28
SAA4	0.0006	0.24	0.0147	0.30
C6orf223	0.0004	0.22	0.0404	0.36
C14orf64	<0.0001	0.22	0.2329	0.61
LTF	0.0009	0.20	0.0052	0.16
HOXC4	0.0001	0.19	0.2906	0.58
SAA2	0.0008	0.10	0.0002	0.10
SAA1	0.0005	0.10	<0.0001	0.11
AHSP	0.0026	0.09	0.3306	0.47

[1] The table lists all genes significantly impacted by smoking (uncorrected p<0.005, fold change > 1.5) as assessed by RNA-Seq for n=5 nonsmokers an n=6 smokers. n=12 nonsmokers and n=12 healthy smokers were assessed by microarray and the p value and fold change for the probeset with largest fold change was returned.

Additional Figure Legends

Figure S1. Verification of RNA-Seq expression levels by TaqMan real time PCR. For 11 genes chosen to represent a wide range of RPKM values, the mRNA level by TaqMan was determined for n=5 cDNA samples of small airway epithelium from nonsmokers. Analysis used the relative quantity compared to rRNA reference (ΔCt method) with duplicates of two dilutions of each cDNA. The mean ΔCt for each gene was multiplied by a constant to make the axes comparable and plotted against the RPKM.

Figure S2. Examples of smoking-responsive genes in the small airway epithelium as assessed by RNA-Seq. Shown are the RNA-Seq sequence alignments with the read coverage depth plotted for 3 healthy nonsmokers and 3 healthy smokers for the genes most affected by smoking in a different fashion. RNA-Seq mapped reads plotted with Partek Genomics Suite software version 6.5, Partek Inc., St. Louis, MO, USA. **A-D.** Examples of genes with the largest absolute increase in expression of nonsmokers compared to smokers. **A.** C20orf114; **B.** ALDH3A1; **C.** FTL; and **D.** TFF3. **E-H**. Examples of genes with the largest absolute decrease in expression of nonsmokers compared to smokers. **E.** SCGB1A1; **F.** SCGB3A1; **G.** RPLP1; and **H.** CRIP1. **I-L**. Examples of genes whose expression is low in nonsmokers, but increases with smoking. **I.** AKR1B10; **J.** CABYR; **K.** SPP1; and **L.** CYP1B1. **M-P.** Examples of genes whose expression is low in nonsmokers and decreases further with smoking. **M.** LYPD2; **N.** SAA4; **O.** ERP27; and **P.** LYNX1. See Table 7 for complete list with smoker to nonsmoker absolute change and fold-change.

Figure S1

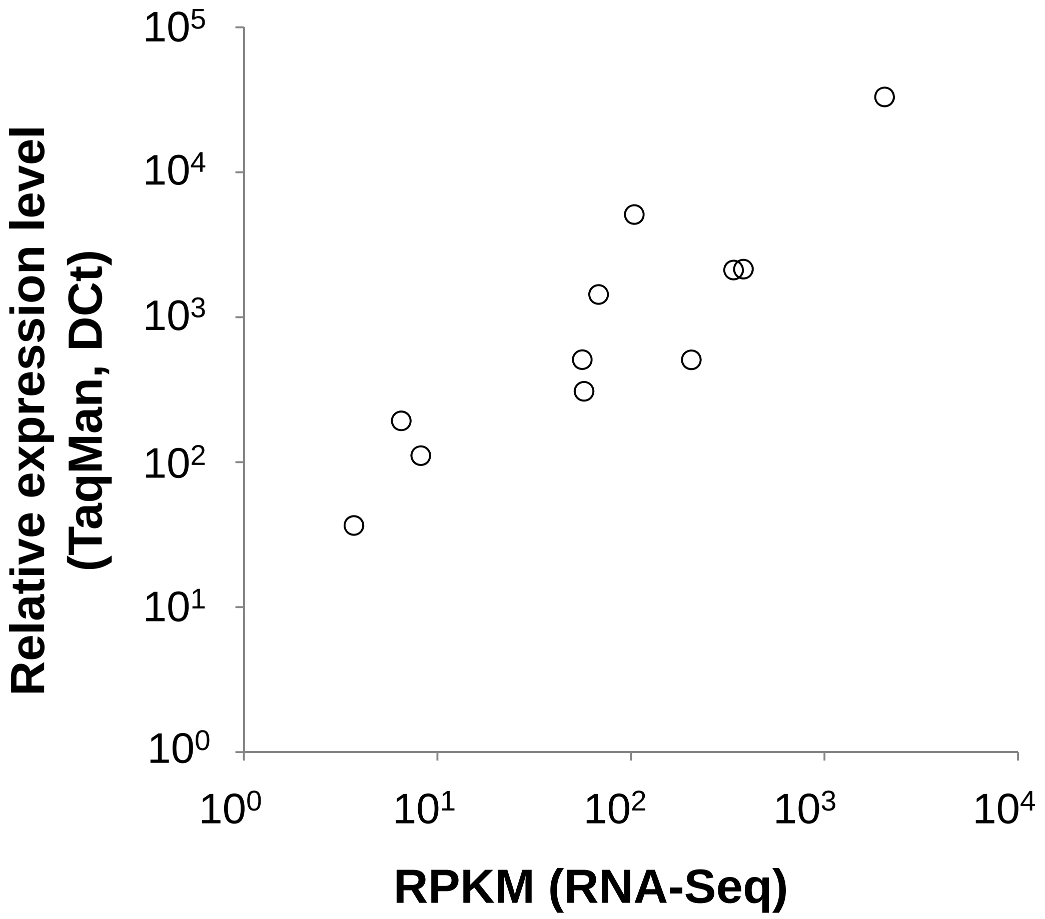

Relative expression level (TaqMan, DCt)
10^5
10^4
10^3
10^2
10^1
10^0
10^0
10^1
10^2
10^3
10^4
RPKM (RNA-Seq)

Figure S2 A-F

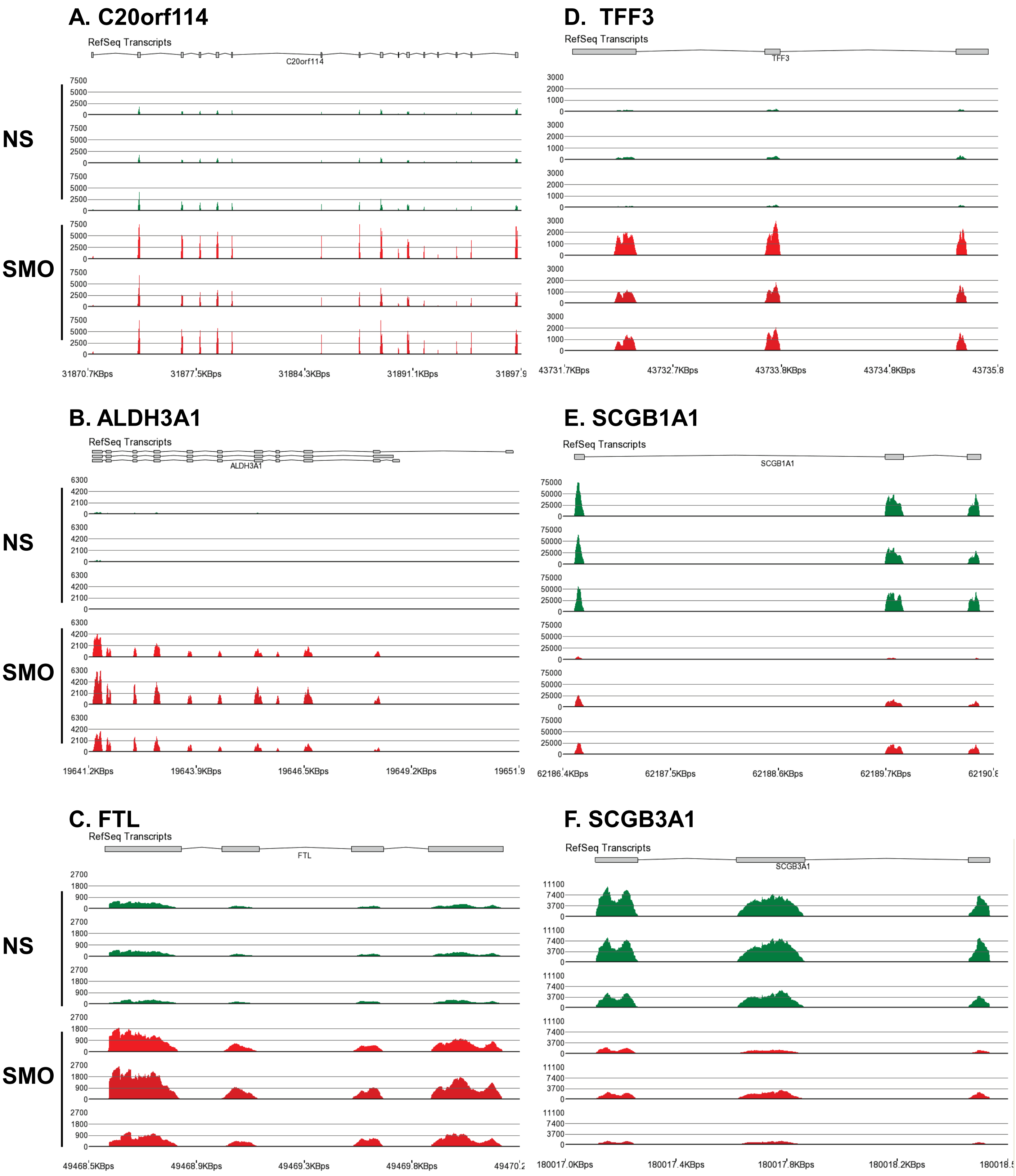
A. C20orf114
RefSeq Transcripts
C20orf114
NS
SMO
7500
5000
2500
0
31870.7KBps
31877.5KBps
31884.3KBps
31891.1KBps
31897.9
B. ALDH3A1
RefSeq Transcripts
ALDH3A1
6300
4200
2100
0
19641.2KBps
19643.9KBps
19646.5KBps
19649.2KBps
19651.9
C. FTL
RefSeq Transcripts
FTL
2700
1800
900
0
49468.5KBps
49468.9KBps
49469.3KBps
49469.8KBps
49470.2
D. TFF3
RefSeq Transcripts
TFF3
3000
2000
1000
0
43731.7KBps
43732.7KBps
43733.8KBps
43734.8KBps
43735.8
E. SCGB1A1
RefSeq Transcripts
SCGB1A1
75000
50000
25000
0
62186.4KBps
62187.5KBps
62188.6KBps
62189.7KBps
F. SCGB3A1
RefSeq Transcripts
SCGB3A1
11100
7400
3700
0
180017.0KBps
180017.4KBps
180017.8KBps
180018.2KBps

Figure S2 G-L

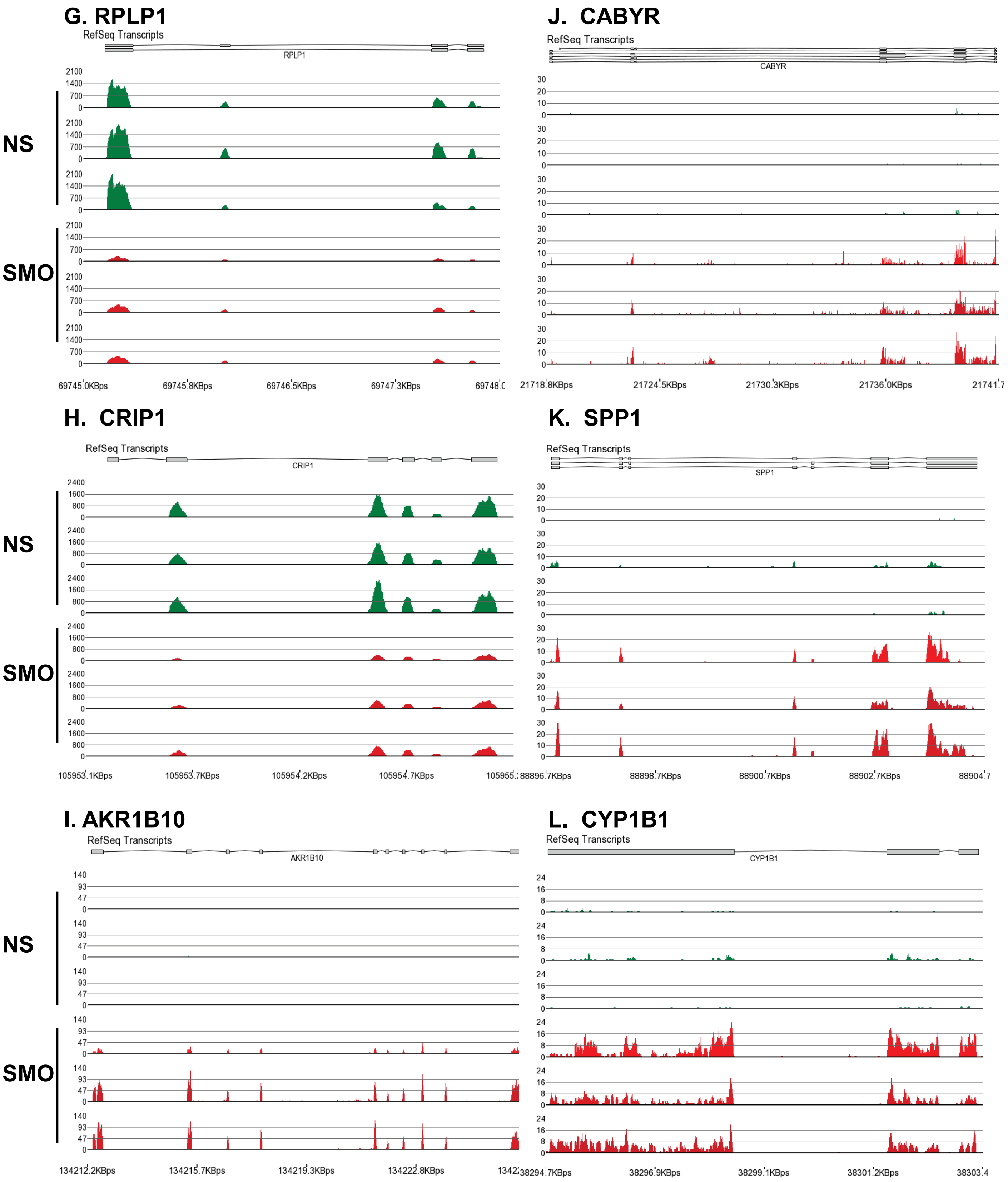
G. RPLP1
RefSeq Transcripts
RPLP1
NS
SMO
2100
1400
700
0
69745.0KBps
69745.8KBps
69746.5KBps
69747.3KBps
J. CABYR
RefSeq Transcripts
CABYR
30
20
10
0
21718.8KBps
21724.5KBps
21730.3KBps
21736.0KBps
H. CRIP1
RefSeq Transcripts
CRIP1
NS
SMO
2400
1600
800
0
105953.1KBps
105953.7KBps
105954.2KBps
105954.7KBps
K. SPP1
RefSeq Transcripts
SPP1
30
20
10
0
88896.7KBps
88898.7KBps
88900.7KBps
88902.7KBps
I. AKR1B10
RefSeq Transcripts
AKR1B10
NS
SMO
140
93
47
0
134212.2KBps
134215.7KBps
134219.3KBps
134222.8KBps
L. CYP1B1
RefSeq Transcripts
CYP1B1
24
16
8
0
38294.7KBps
38296.9KBps
38299.1KBps
38301.2KBps

Figure S2 M-P

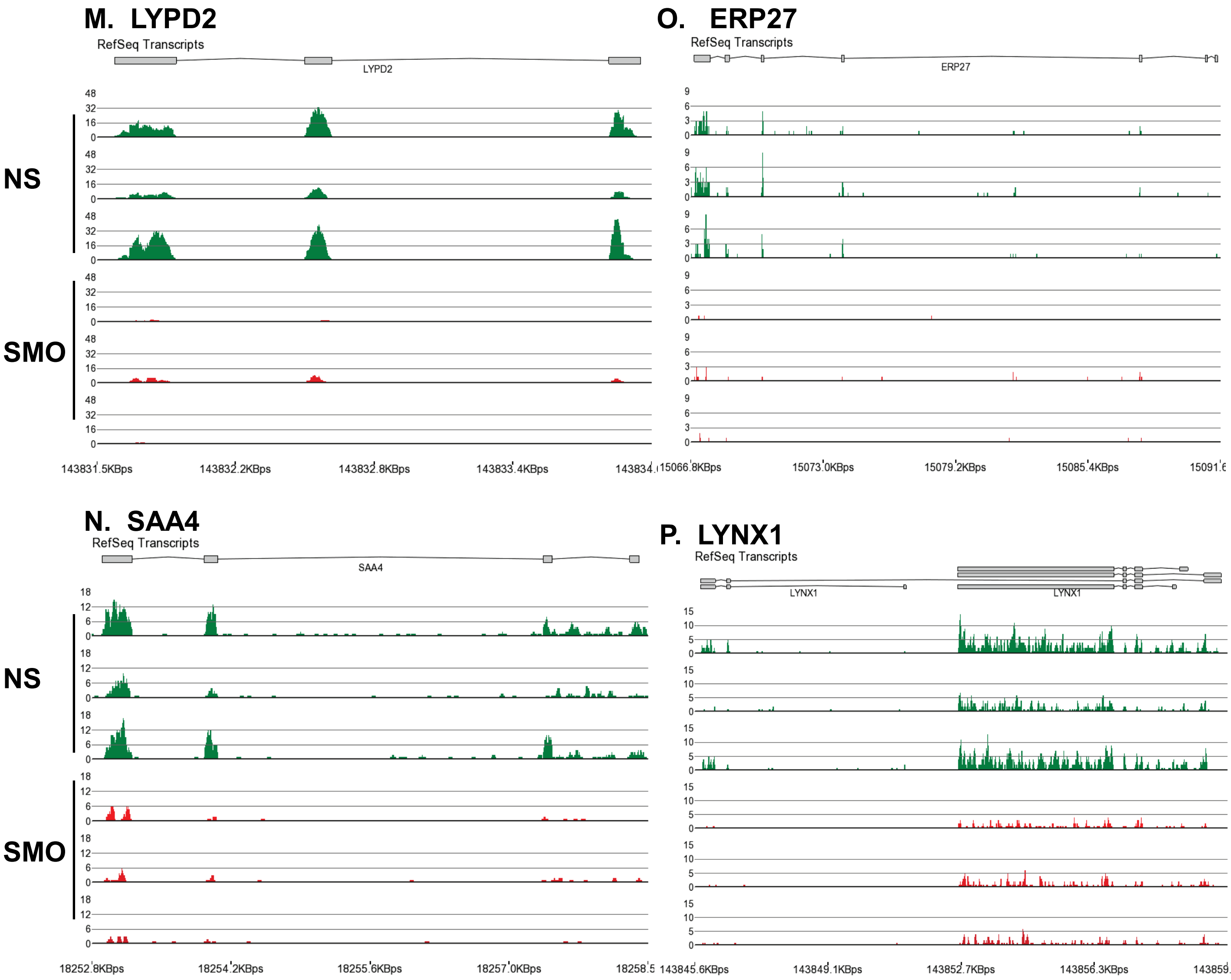
M. LYPD2
RefSeq Transcripts
LYPD2
NS
SMO
48
32
16
0
143831.5KBps
143832.2KBps
143832.8KBps
143833.4KBps
O. ERP27
RefSeq Transcripts
ERP27
9
6
3
0
15066.8KBps
15073.0KBps
15079.2KBps
15085.4KBps
N. SAA4
RefSeq Transcripts
SAA4
18
12
6
0
18252.8KBps
18254.2KBps
18255.6KBps
18257.0KBps
P. LYNX1
RefSeq Transcripts
LYNX1
LYNX1
15
10
5
0
143845.6KBps
143849.1KBps
143852.7KBps
143856.3KBps
